# Identification of biomarkers of brown adipose tissue aging highlights the role of dysfunctional energy and nucleotide metabolism pathways

**DOI:** 10.1038/s41598-021-99362-1

**Published:** 2021-10-07

**Authors:** Carola Mancini, Sabrina Gohlke, Francisco Garcia-Carrizo, Vyacheslav Zagoriy, Heike Stephanowitz, Tim J. Schulz

**Affiliations:** 1 Department of Adipocyte Development and Nutrition, German Institute of Human Nutrition Potsdam-Rehbrücke, 114-116 Arthur-Scheunert-Allee, 14558 Nuthetal, Germany; 2 German Center for Diabetes Research (DZD), München-Neuherberg, Germany; 3 metaSysX GmbH, Potsdam-Golm, Germany; 4 Leibniz-Forschungsinstitut für Molekulare Pharmakologie (FMP), Berlin, Germany; 5 Institute of Nutritional Science, University of Potsdam, Potsdam-Rehbrücke, Nuthetal, Germany

**Keywords:** Ageing, Metabolism, Fat metabolism, Metabolic diseases, Metabolomics

## Abstract

Brown adipose tissue function declines during aging and may contribute to the onset of metabolic disorders such as diabetes and obesity. Only limited understanding of the mechanisms leading to the metabolic impairment of brown adipocytes during aging exists. To this end, interscapular brown adipose tissue samples were collected from young and aged mice for quantification of differential gene expression and metabolite levels. To identify potential processes involved in brown adipocyte dysfunction, metabolite concentrations were correlated to aging and significantly changed candidates were subsequently integrated with a non-targeted proteomic dataset and gene expression analyses. Our results include novel age-dependent correlations of polar intermediates in brown adipose tissue. Identified metabolites clustered around three biochemical processes, specifically energy metabolism, nucleotide metabolism and vitamin metabolism. One mechanism of brown adipose tissue dysfunction may be linked to mast cell activity, and we identify increased histamine levels in aged brown fat as a potential biomarker. In addition, alterations of genes involved in synthesis and degradation of many metabolites were mainly observed in the mature brown adipocyte fraction as opposed to the stromal vascular fraction. These findings may provide novel insights on the molecular mechanisms contributing to the impaired thermogenesis of brown adipocytes during aging.

## Introduction

Brown adipose tissue (BAT) is responsible for thermoregulation in mammalians, allowing the organism to maintain a constant body temperature independent of the environment. The thermogenic capacity of this tissue is due to its high mitochondrial content and unique expression of Uncoupling protein-1 (UCP1). Localized in the inner mitochondrial membrane, UCP1 uncouples the mitochondrial proton gradient generated by oxidative phosphorylation from ATP-synthesis, instead enabling heat production. This process is called non-shivering or adaptive thermogenesis^1,2^. Although *Ucp1* gene expression is highly selective for brown adipocytes, the emergence of brown-like adipocytes in response to adrenergic stimulation by prolonged cold-exposure or treatment with selective β3-adrenergic receptor agonists has been reported in white adipose tissue as well, leading to browning of this type of adipose tissue^3–5^. BAT thermogenic function and energy expenditure capacity are negatively correlated with aging^6–9^. In humans, a decreased incidence of active BAT has been found in elderly subjects^4^. A similar age-dependent metabolic dysfunction of BAT has also been demonstrated in rodent models^10^. Increased age results in a loss of BAT mass and activity, corresponding to an impairment of the thermogenic response and a detrimental effect on brown adipocyte formation and function^11^. Several studies suggest that age-related thermogenic defects of brown fat may be due to cell-autonomous alterations, such as mitochondrial dysfunction and reduced UCP1 activity in aged brown adipocytes^12^. Additionally, changes of the local microenvironment of brown adipocytes occur during aging, leading to a reduction of sympathetic innervation and altered endocrine signals that may attenuate thermogenesis^13^. Our own previous work also reported altered lipid metabolism in aged BAT which was accompanied by aberrant lipid utilization within brown adipocytes as well as bioactive lipid signaling molecules which could inhibit metabolic activity of brown adipocytes^14,15^. Given its function as a thermogenic organ, BAT has been proposed as a target for potential treatment strategies of age-related metabolic conditions, such as obesity and type 2 diabetes. To exert their energy expenditure-function, brown adipocytes need to regulate UCP1’s thermogenic activity efficiently in order to avoid excessive loss of chemical energy and to maintain energy homeostasis. UCP1-mediated proton conductance can be inhibited by di- and triphospho-purine nucleotides (ADP, ATP, GDP, GTP)^16–18^. It is also activated by lipolytic release of free fatty acids^19,20^, thereby constituting a regulatory metabolite system of the thermogenic response. In addition, β-adrenergic stimulation has been linked to alterations of cytosolic free purine nucleotide concentrations^21^, in particular regarding the inhibitory effect of purine nucleotides on UCP1, which may be attenuated in brown adipocytes either by alterations of purine concentrations or by shifting the balance from free towards magnesium-complexed nucleotides, as the latter do not inhibit UCP1^22,23^. This evidence suggests that changes in concentration of different metabolites in BAT may contribute to the regulation of thermogenesis.

In order to identify new molecular mediators involved in the progressive loss of thermogenic brown adipocytes during aging, BAT collected from mice at different ages was used for metabolomic, proteomic and gene expression analyses. Three polar metabolite-gene clusters related to energy, vitamin and nucleotide metabolism were identified to be affected by aging. Genes involved in nucleotide metabolism were more highly expressed in adipocytes compared to cells of the adipose tissue stromal vascular fraction (SVF), and age-related alterations were only observed in brown, but not in white adipose tissue, suggesting that these changes may be suitable biomarkers of the functional decline in brown adipocytes during aging. In summary, our findings show that changes in concentrations of nucleotide metabolites occur during BAT-aging, and they may contribute to the detrimental effect of age on brown adipocyte function.

## Results

### Identification of potential metabolic biomarkers of BAT-aging

**Figure 1:**
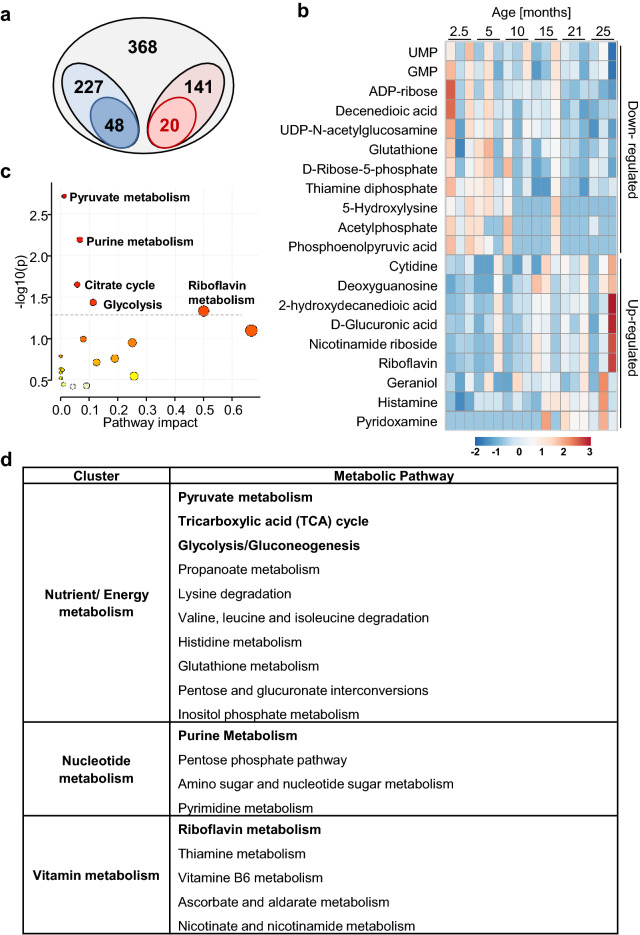
Changes of polar metabolites during BAT-aging. (**a**) Venn diagram representing metabolites from BAT samples that were collected from animals aged 2.5, 5, 10, 15, 21, and 25 months (n = 3/ age-group). All metabolites were detected by LC–MS and GC–MS metabolomic analysis. 368 compounds were detected (light grey) and grouped into non-polar lipid species (227 candidates; light blue) and polar metabolites (141 candidates; light red). For each group, clusters of metabolites with significant correlation to aging across the age-groups were identified, corresponding to 48 non-polar lipid species (dark blue) and 20 polar metabolites (dark red). (**b**) Heatmap representing concentrations of the 20 individual polar metabolites that were significantly correlated to BAT-aging in the individual animals from each age group. (**c**) Pathway enrichment analysis for the 20 polar metabolites with significant age-related correlation using MetaboAnalyst software. Grey line indicates formal threshold of significant enrichment. (**d**) Summary of all pathways identified from the enriched polar metabolites, including pathways with significant enrichment (bold; p < 0.05), after grouping into three functionally related sub-clusters, i.e. (1) nutrient/ energy metabolism, (2) nucleotide metabolism, and (3) vitamin metabolism.

To identify novel biomarkers and mechanisms of defective BAT function during aging, a comparative metabolomic analysis by mass spectrometry was used. Data analysis of male mice of the C57BL/6-J strain resulted in a total of 368 identified metabolites in BAT samples of five different age groups, ranging from 2.5 to 25 months of age (Fig. 1a). A broad variety of metabolites was detected, including 227 non-polar and 141 polar metabolites which contained intermediates belonging to different metabolic pathways. When analyzing changes of metabolites in BAT from each age group, we identified a total of 48 lipids and 20 polar metabolites with significant age-dependent correlations (Fig. 1a). The subset of non-polar, lipid metabolites were further analyzed in a previous study which identified several lipid species as functional biomarkers of BAT-aging, including a sphingolipid, Sphingosine-1-phopshate, and an isoprenoid-derived lipid class, the dolichols, which exerted regulatory effects on brown adipocyte formation and function^14^. Polar metabolites showing age-dependent correlations in BAT were further investigated here, revealing negative or positive correlations for 11 and 9 metabolites, respectively (Fig. 1b). Despite this limited number, pathway analysis of these 20 age-regulated candidates was performed using the software MetaboAnalyst to broadly annotate the dataset for metabolic processes involving these metabolites. This allowed us to assign the metabolites to three pathway clusters, i.e. being related to either nutrient/energy metabolism, or nucleotide metabolism, or vitamin metabolism (Fig. 1c,d). The pathways driving the cluster of nutrient and energy metabolism were annotated as pyruvate metabolism, glycolysis/gluconeogenesis, and the tricarboxylic acid cycle (TCA). Altogether, this observation confirms that BAT, like many other tissues, experiences mitochondrial dysfunction and defective energy metabolism during aging. The cluster of nucleotide metabolism was mainly represented by enrichment of metabolites within the purine metabolism pathway with concomitant identification of pyrimidine metabolism. An additional third cluster was mostly represented by metabolic processes related to vitamins, in particular to those belonging to the group of B-vitamins (riboflavin, thiamine, pyridoxine and nicotinamide).

**Figure 2:**
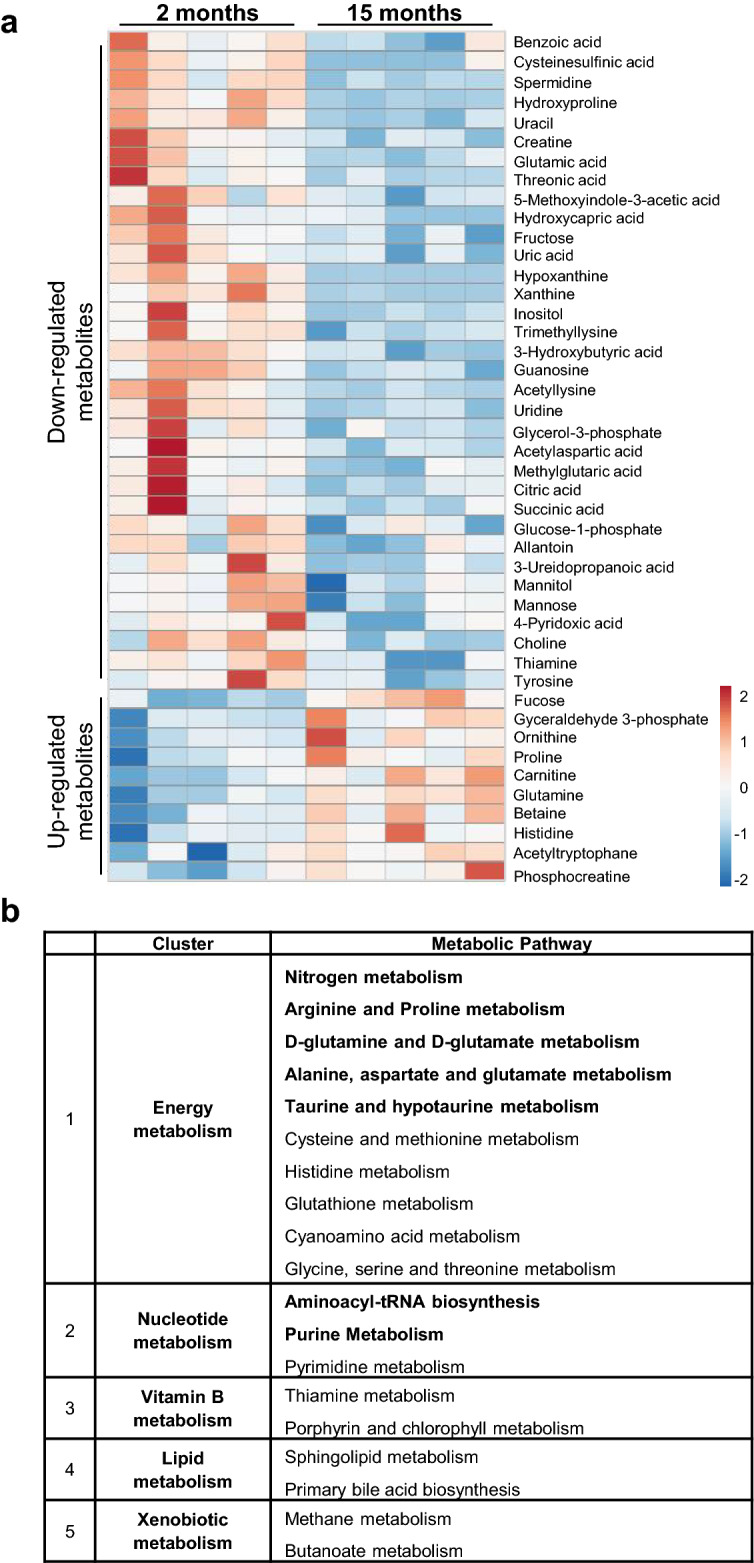
Age-related changes in plasma metabolites reflect effects on purine metabolism in aged BAT. (**a**) Heatmap depicting plasma metabolite concentrations comparing plasma samples from young (2 months) to aged (15 months) mice that are significantly (p < 0.05) different between both age groups (n = 5 / group). All metabolites were detected by LC–MS and GC–MS metabolomic analysis. (**b**) Pathway identification analysis for polar and non-polar plasma metabolites with age-related changes using MetaboAnalyst software. Metabolites were selected based on significant (p < 0.05) differences between age groups. Tabular summary of all pathways identified from plasma metabolites, including pathways with significant enrichment (bold; p < 0.05), which were subsequently grouped into functionally related sub-clusters, i.e. (1) nutrient/ energy metabolism, (2) nucleotide metabolism, (3) vitamin metabolism, (4) lipid metabolism and (5) xenobiotic metabolism.

To correlate systemic metabolomic changes in relation to aging in BAT, a metabolomic assessment of plasma samples collected from young and aged mice was performed. Among the detected compounds, 34 metabolites were age-depleted and 10 metabolites were significantly up-regulated in aged mice (Fig. 2a). To annotate each metabolite to metabolic processes, a pathway analysis similar to the analysis of BAT was performed, revealing only limited overlap with metabolic clusters identified in aging BAT (Fig. 2b, Supplementary Table S1). While individual metabolites were not overlapping between analyses of BAT and plasma, pathways particularly linked to nucleotide, i.e. purine and pyrimidine, metabolism and also energy metabolism and B-vitamin metabolism were identified. In summary, age-dependent regulation of pathways involved in energy, nucleotide and B-vitamin metabolism in BAT were reflected partially by age-related alterations in circulating metabolite levels.

### Histamine indicates negative effects of BAT resident mast cells on energy metabolism

**Figure 3:**
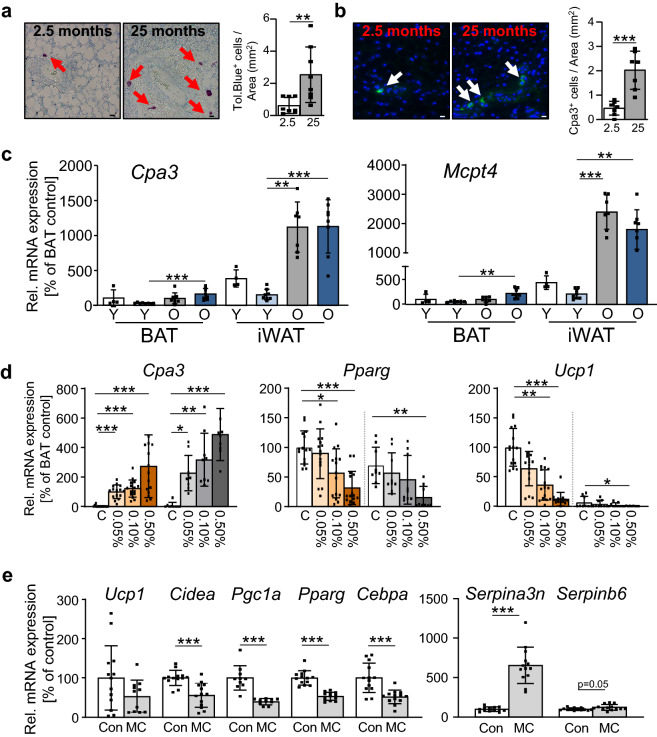
Aging results in mast cell accumulation which impairs brown adipogenesis. (**a**) Toluidine blue staining of BAT-sections of young (2.5 months, left panel; n = 7) and aged mice (25 months; middle panel, n = 7) and subsequent cell count-based quantification (right panel) of stained mast cells (red arrows) normalized to total section area using sections of BAT. For each animal, 1–2 independent tissue sections per animal were assessed. Images were collected at 600-fold magnification (scale bar: 10 µm). (**b**) Immunofluorescence analysis of cells expressing mast cell marker carboxypeptidase A3 (CPA3) in BAT-sections of young (2.5 months, left panel; n = 8) and aged mice (25 months; middle panel, n = 8) and subsequent cell count-based quantification (right panel) of stained mast cells (green signal, white arrows) normalized to total section area using sections of BAT. For each animal, 1–2 independent tissue sections per animal were assessed. Images were collected at 600-fold magnification (scale bar: 10 µm). (**c**) mRNA levels of mast cell markers *Cpa3* and *Mcpt4* in BAT (left) and iWAT (right) of young mice maintained at room temperature (RT; white bars) or of young mice after 7 days of cold exposure (light blue) were compared to mice aged 25 months at RT (grey bars) or 25-month old mice after 7-day cold exposure (dark blue). Data are depicted as mean ± standard deviation (SD; n = 4 for 2.5 months BAT and iWAT; n = 8 for all cold exposed tissues and aged BAT)-8 mice; n = 7 for aged iWAT). (**d**) mRNA expression of *Cpa3*, *Pparg* and *Ucp1* of primary brown (left, orange) and white (right, grey) adipose tissue-derived progenitors co-seeded with 0.05, 0.1 or 0.5% of p815-mast cells compared to control cultures without masts cells (control; C) after 10 days of differentiation (n = 15, from 3 independent repeat-experiments for BAT; n = 9 from 2 independent repeat experiments for iWAT). (**e**) mRNA expression of brown adipocyte marker genes *Ucp1*, *Cidea*, *Ppargc1a*, *Pparg*, *Cebpa* and of mast cell protease inhibitors *Serpina3n* and *Serpin6b* in in vitro differentiated brown adipocytes exposed to control conditioned media (white) or mast cell-conditioned media (light grey) for 24 h (n = 12 for *Ppargc1a* from 2 independent experiments; n = 15 for all other genes, from 3 independent repeat-experiments). All data (see Suppl. Table S5 for full gene names) are depicted as mean ± standard deviation (SD); *p < 0.05, **p < 0.01, ***p < 0.001 using a nonparametric Kruskal–Wallis test for multiple comparisons of panels with multiple BAT- or iWAT-samples, respectively, and non-parametric Mann–Whitney test for panels with pairwise comparisons.

To specifically investigate the age-dependent effects on identified metabolites, time-dependent correlation analyses for single metabolites were performed, revealing that energy metabolism intermediates related to pyruvate metabolism, including acetylphosphate and phosphoenolpyruvic acid, were negatively correlated with BAT-aging, as well as amino acid-metabolites 5-hydroxy lysine and glutathione. Other polar intermediates, including a glucose-derived metabolite, D-glucuronic acid, showed a positive correlation with aging in BAT (Supplementary Fig. S1, Supplementary Table S2). While nutrient/energy metabolism intermediates could reflect metabolic or mitochondrial dysfunction, which is well-documented in many tissues during aging, the positive correlation of histamine with BAT-aging caught our attention. This metabolite was significantly enriched in aged BAT and the amino acid histidine, the precursor of histamine synthesis, was also significantly increased in aged plasma samples. We therefore decided to more closely investigate histamine as a candidate for a potential functional biomarker of aging in BAT. Mast cells, the main source of histamine, have previously been linked to inhibition of thermogenesis in brown adipocytes, although aside from limited data, published studies predominantly address browning of WAT rather than the effect of Mast cells on classical depots of BAT^24,25^. We therefore hypothesized that enrichment of mast cells in aged BAT may contribute to its age-related dysfunction. Indeed, a histological assessment revealed that mast cells were enriched in BAT of aged mice (Fig. 3a and b). Moreover, expression of mast cell marker genes carboxypeptidase A3, *Cpa3*, and mast cell protease 4, *Mcpt4*, was measured in BAT and inguinal white adipose tissue (iWAT) of young, 2.5 months-, and aged, 25 months-old mice before and after a 7-day cold exposure. These analyses showed that aging alone tended to increase expression of mast cell markers in iWAT but not BAT, while this effect of increased expression of both genes was significant between both tissues after cold exposure (Fig. 3c). To functionally link mast cell activity to brown and white adipocyte differentiation, we next performed co-culture assays where primary pre-adipocytes isolated from brown and white adipose tissue by flow cytometry were differentiated in the presence of increasing numbers cells of the mast cell line p815 ranging from 0.05 – 0.5% of mast cells as proportion of total cells in each culture well^26^. While expression of mast cell marker *Cpa3* correlated with the increasing percentages of mast cells in the co-culture, expression of *Pparg* was reduced in brown adipogenic co-cultures at 0.1 and 0.5% and white adipogenic co-cultures at the highest percentage of p815 mast cells. A strong inverse correlation between the percentage of mast cells in the culture and expression of brown adipogenic marker *Ucp1* was found with a virtually absent expression in co-cultures with the highest mast cell proportion (Fig. 3d). To rule out dilution effects due to proliferation of mast cells, we also cultivated differentiated brown adipocytes in cell culture media previously conditioned by p815 mast cells. Recapitulating the findings from the co-cultures, treatment with such media resulted in a rapid reduction of expression of brown adipogenic markers and induction of serpin genes, *Serpina3n* and *Serpinb6*, which are inhibitors of mast cell proteases^27^, suggesting that a paracrine crosstalk between the two cell types, through factors released from mast cells, may affect brown adipocyte function (Fig. 3e). In summary, these observations suggest that the age-related increase in histamine may act as a biomarker of increased mast cell accumulation in BAT, which in turn could exert inhibitory effects on brown adipocyte function.

### Altered nucleotide metabolism as biomarker of brown adipose tissue-aging

**Figure 4:**
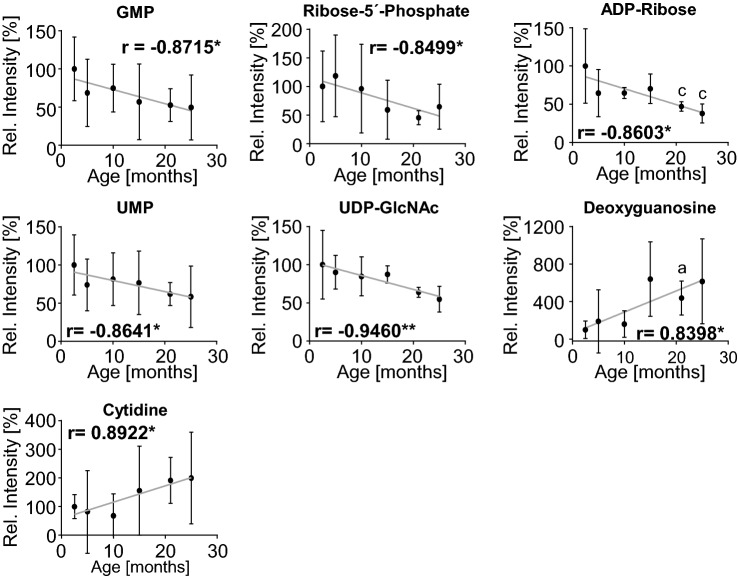
Polar metabolites involved in nucleotide metabolism correlate with BAT aging. Spearman correlations of individual polar metabolites linked to nucleotide metabolism that are significantly associated with aging (from left to right, top to bottom): Guanosine monophosphate (GMP), ribose-5´-phosphate, adenosine diphosphate-ribose (ADP-ribose), uridine monophosphate (UMP), uridine diphosphate N-acetylglucosamine (UDP-GlcNAc), deoxyguanosine, and cytidine. Correlation coefficients (r) for individual metabolites were calculated to visualize age-dependent correlations of metabolic species. Individual peak intensities of all age groups were normalized to intensities measured in the 2.5 months control group. All data are depicted as mean ± SD (n = 3 for all groups); * p < 0.05 indicating significant correlation across all age groups; ^a, b, c, d^ p < 0.05 comparing relative intensity of the indicated age group compared to (a) 2.5 months, (b) 5 months, (c) 10 months or (d) 15 months, respectively, using two-tailed unpaired t-test.

The second cluster of aging-sensitive correlations in BAT, nucleotide metabolism, was driven by several purine- and pyrimidine-linked metabolites, i.e. guanosine monophosphate (GMP), ribose-5´-phosphate, adenosine diphosphate-ribose (ADP-ribose), uridine monophosphate (UMP) and UDP-N-acetyl glucosamine, which correlated inversely with age. Moreover, one purine nucleotide, deoxyguanosine, and one pyrimidine nucleoside, cytidine, were found to be positively correlated with BAT-aging (Fig. 4, Supplementary Table S3). Age-dependent regulation of additional purine intermediates was observed when directly comparing metabolite concentrations for each age group to the youngest, 2.5-month old reference group, showing guanosine, inosine, and the purinogenic amino acid L-glutamine to be significantly elevated in the old groups (Supplementary Fig. S2, Supplementary Table S3). In addition, four intermediates related to B-vitamin metabolism were detected using this analysis, thiamine pyrophosphate (TPP), which was negatively correlated to BAT-aging, and riboflavin, pyridoxamine and nicotinamide riboside, which showed an induction in the aged groups compared to 2.5-month old BAT (Supplementary Fig. S2, Supplementary Table S4).

**Figure 5:**
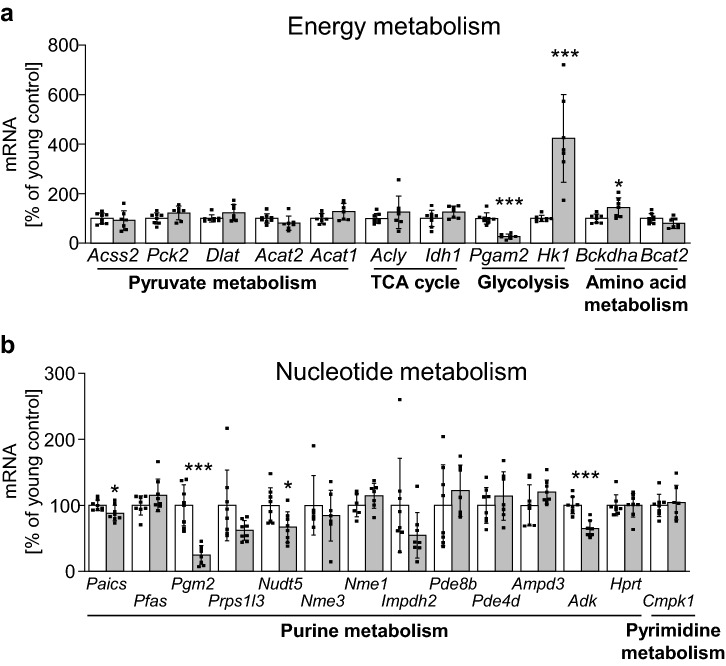
mRNA expression of biosynthesis and degradation genes linked to metabolites identified in aged BAT. (**a**) mRNA levels of nutrient/ energy metabolism genes (see Suppl. Table S5 for full gene names) comparing BAT samples of young (2.5 months; n = 8; white bars) and aged (15 months; n = 7; grey bars) mice. (**b**) mRNA levels of nucleotide metabolism genes comparing BAT samples of young (2.5 months; n = 8; white bars) and old (15 months; n = 8; grey bars) mice. All genes are organized according to pathway enrichment in the proteome analysis of the same age groups (Table 1). Data are expressed as percentage of young (2.5 months) control. Data are shown as mean ± SD; *p < 0.05; **p < 0.01; ***p < 0.001 using non-parametric Mann–Whitney test.

**Figure 6:**
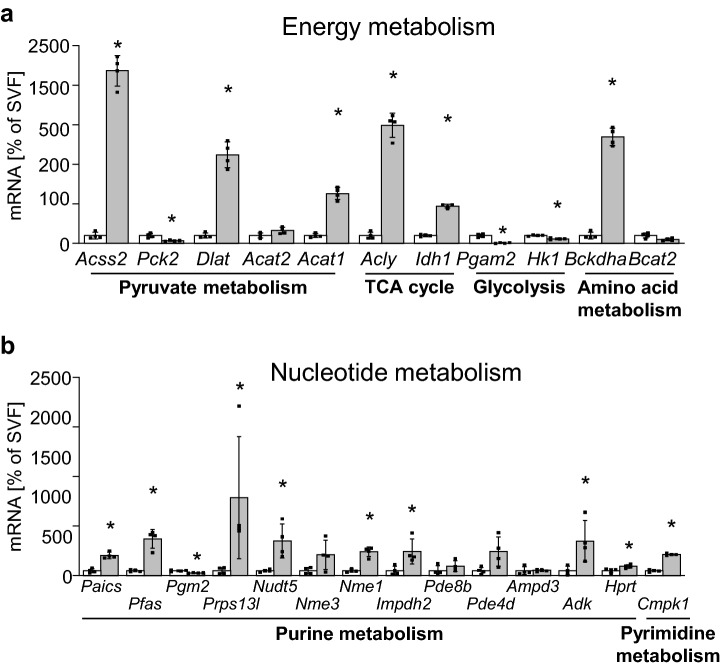
Expression of marker genes is enriched in mature adipocyte fraction. (**a**) mRNA levels of nutrient/ energy metabolism genes and of nucleotide metabolism genes (**b**) assessed in SVF (white bars) and mature adipocytes (grey bars) isolated from BAT of 2.5-months old mice. Genes (see Suppl. Table S5 for full gene names) are organized according to pathway enrichment in the proteome analysis of the same age groups (Table 1). For each gene, data are expressed as percentage of BAT-derived SVF. Data are shown as mean ± SD (n = 4 for all groups); *p < 0.05; assessed using non-parametric Mann–Whitney test.

**Figure 7:**
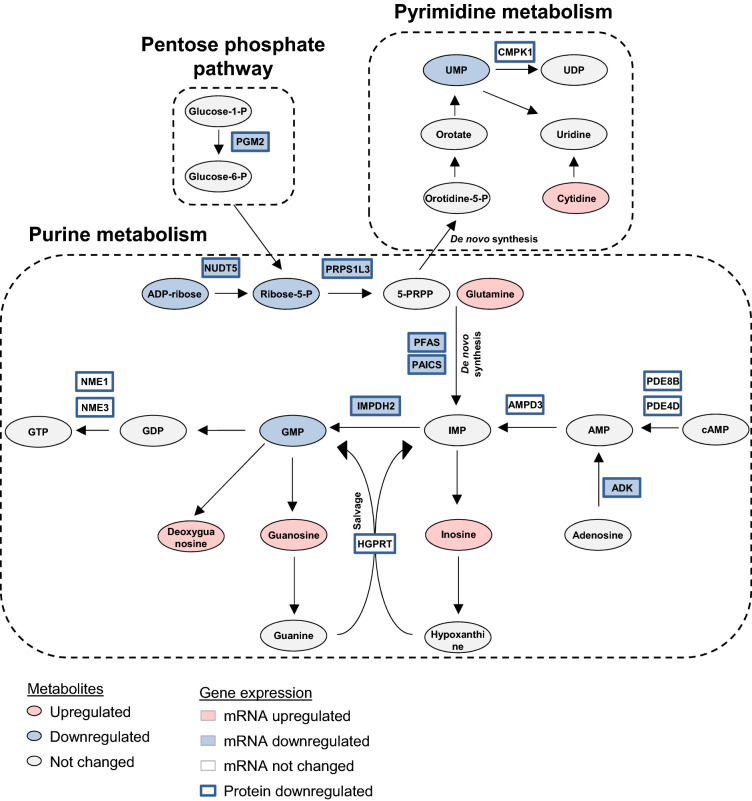
Concerted age-dependent regulation of nucleotide metabolism in BAT. Metabolic network representing nucleotide metabolism and integrating age-dependent regulation observed in metabolomic, proteomic and mRNA expression data analyses. Dashed line-boxes depict different metabolic branches of nucleotide metabolism: purine metabolism, pyrimidine metabolism and pentose phosphate pathway. Each oval represents a metabolite and each box represents a gene/protein. As shown in the legend, color-coding is provided for metabolites, genes and proteins enriched (red) or depleted (blue) during BAT-aging. Metabolites whose concentration was not affected by aging in BAT are shown in grey. Color-coding of metabolites is based on the log2 fold change (log2FC) values calculated in comparison to the young, 2.5-months control group (Supplementary Table S2). For each protein and gene, age-dependent regulation is represented by the same color-coding; genes whose mRNA was not changed during BAT-aging are depicted in white boxes. Proteomic changes are based on heavy/light ratios comparing young versus aged mice (Table 1).

To investigate whether the metabolic pathways identified through the metabolite analysis were also altered on the level of gene expression linked to these substances, we screened a proteomic dataset of aging BAT that we had previously generated by 18O-labeling mass spectrometry for candidates involved in enzymatic reactions of the three identified metabolic clusters^14^. Proteins were annotated depending on their pathway-association within the metabolic process and, for each protein, the relative heavy/light (H/L) ratio of the 18O-labeling analysis was calculated from the intensity-weighted average of all detected peptide ratios, with values of < 1.0 indicating age-dependent down-regulation and H/L ratios > 2.5 depicting age-enriched proteins^14^. This analysis revealed a prominent age-dependent down-regulation of most detected proteins within all three pathway clusters, where the highest number of age-depleted candidates was related to purine metabolism (Table 1). Of note, only two proteins were enriched in BAT of aged mice, the glycolysis enzymes hexokinase 1 (HK1) and phosphoglycerate mutase 2 (PGAM2). To verify these observations, we also tested mRNA expression of these genes linked to energy or nucleotide metabolism in BAT and, additionally, in iWAT, comparing 2.5- and 15-months old animals. However, a significant age-dependent up-regulation was only detected for *Hk1*, whereas *Pgam2* was found to be reduced on the mRNA level, and the remaining energy metabolism genes were not differentially expressed (Fig. 5a). In genes linked to nucleotide metabolism, a significant down-regulation of mRNA expression of several enzymes involved in purine metabolism was detected (Fig. 5b), further corroborating the previous findings of age-dependent decrease of those candidates on the proteomic and metabolite level. We also investigated expression of B-vitamin metabolism-related genes, but no changes of transcript levels of these candidates were found in aged BAT (Supplementary Fig. S3). To further assess whether comparable alterations may also occur in aged white fat, a similar set of genes was measured in iWAT, but only sparse and less consistent regulations of genes throughout the different metabolic clusters were detected in this adipose tissue depot (Supplementary Fig. S4). In summary, aging resulted in consistent changes in the expression of enzymes related to nucleotide, and in particular, purine metabolism in aging BAT. Given the heterogenous composition of brown and white adipose tissue, we also evaluated expression of the same set of genes in isolated brown and white adipocytes in comparison to the respective SVF of BAT and iWAT. These analyses revealed that the majority of genes involved in either energy or nucleotide metabolism pathways were significantly enriched in mature brown and white adipocytes compared to the respective SVF. As the stromal vascular fraction will contain all blood cells, but also all non-adipocytes permanently resident within the adipose tissue, these findings suggest that the metabolite changes observed in aged BAT were indeed a consequence of altered metabolic processes in adipocytes rather than stemming from residual blood or SVF cells within the tissue (Fig. 6a, b). Expression of B-vitamin genes was not similarly enriched in brown adipocytes (Supplementary Fig. S5), and mRNA levels of the same set of genes were also less consistently enriched in mature white adipocytes compared to iWAT-derived SVF (Supplementary Fig. S6). To illustrate the concerted age-related regulation of metabolic flux in brown adipose tissue, we graphically integrated metabolomic, proteomic and transcriptional changes in a metabolic network representing the nucleotide metabolism cluster (Fig. 7) and the energy metabolism pathways (Supplementary Fig. S7). Taken together, these data suggest that aging results in changes of BAT metabolism on several levels which could serve to better define biomarkers depicting age-related dysfunction of energy metabolism and thermogenesis in brown adipocytes.

**Table 1:** Age-dependent proteomic regulation of nucleotide metabolism enzymes. Table representing enzymes involved in (1) nutrient/ energy metabolism, (2) nucleotide metabolism and (3) B-vitamin metabolism identified by comparative 18O-labeled proteomic analysis of young and aged BAT (n = 5). Proteins were annotated based on their Uniprot accession number^53^. Identified proteins were clustered depending on their function in the metabolic pathway: (1) pyruvate metabolism, TCA cycle, glycolysis, amino acid metabolism; (2) purine and pyrimidine metabolism; (3) vitamin transport, vitamin B6 metabolism and nicotinamide metabolism. Proteins that were reduced in aged mice were identified based on heavy/light ratios (H/L ratio) < 1, while H/L ratios > 2.5 depicted age-enriched enzymes.

Cluster	Metabolic pathway	Protein name (Uniprot accession no.)	Gene symbol	RefSeq ID	Proteomic H/L ratio
**Energy metabolism**	**Pyruvate metabolism**	Acyl-CoA Synthetase Short Chain Family Member 2 (A2AQN4)	*Acss2*	NM_019811	0.88
		Phosphoenolpyruvate Carboxykinase 2, Mitochondrial (Q8BH04)	*Pck2*	NM_028994	0.76
		Dihydrolipoamide S-Acetyltransferase (Q8BMF4)	*Dlat*	NM_145614	0.95
		Acetyl-CoA Acetyltransferase 2 (Q8CAY6)	*Acat2*	NM_009338	0.76
		Acetyl-CoA Acetyltransferase 1 (Q8QZT1)	*Acat1*	NM_144784	0.94
	**TCA Cycle**	ATP Citrate Lyase (Q91V92)	*Acly*	NM_134037	0.96
		Isocitrate Dehydrogenase 1 (O88844)	*Idh1*	NM_001111320	0.84
	**Glycolysis**	Phosphoglycerate Mutase 2 (O70250)	*Pgam2*	NM_018870	4.12
		Hexokinase 1 (G3UVV4)	*Hk1*	NM_001146100	2.98
	**Amino acid metabolism**	Branched Chain Keto Acid Dehydrogenase E1 Subunit Alpha (Q3U3J1)	*Bckdha*	NM_007533.5	0.89
		Branched Chain Amino Acid Transaminase 2 (D3Z7C8)	*Bcat2*	NM_001243052	0.97
**Nucleotide metabolism**	**Purine metabolism**	Multifunctional protein ADE2 (Q9DCL9)	*Paics*	NM_001356971	0.52
		Phosphoribosylformylglycinamidine synthase (Q5SUR0)	*Pfas*	NM_001159519	0.54
		Phosphoglucomutase-2 (Q7TSV4)	*Pgm2*	NM_025700	0.70
		Phosphoribosyl pyrophosphate synthetase 1-like 3 (G3UXL2)	*Prps1l3*	NM_001037746	0.75
		ADP-sugar pyrophosphatase (Nudix Hydrolase 5) (Q9JKX6)	*Nudt5*	NM_016918	0.80
		Nucleoside Diphosphate Kinase 3 (Q9WV85)	*Nme3*	NM_019730	0.38
		Nucleoside Diphosphate Kinase 1 (P15532)	*Nme1*	NM_008704	0.43
		Inosine-5'-monophosphate dehydrogenase 2 (P24547)	*Impdh2*	NM_001378921	0.90
		Phosphodiesterase 8B (P15532)	*Pde8b*	NM_001170669	0.11
		Phosphodiesterase 4D (F6QFD1)	*Pde4d*	NM_011056	0.57
		AMP deaminase 3 (O08739)	*Ampd3*	NM_001276301	0.83
		Adenosine kinase (P55264)	*Adk*	NM_134079	0.93
		Hypoxanthine Phosphoribosyltransferase 1 (P00493)	*Hprt*	NM_013556	0.59
	**Pyrimidine metabolism**	Cytidine/Uridine Monophosphate Kinase 1 (Q9DBP5)	*Cmpk1*	NM_025647	0.83
**Vitamin metabolism**	**Vitamin transport**	Apolipoprotein A1 (Q00623)	*Apoa1*	NM_009692	0.66
		Apolipoprotein A4 (P06728)	*Apoa4*	NM_007468	0.45
	**Vitamin B6 metabolism**	Pyridoxamine 5'-Phosphate Oxidase (Q91XF0)	*Pnpo*	NM_146026	0.70
	**Nicotinamide metabolism**	NAD Kinase 2, Mitochondrial (Q8C5H8)	*Nadk2*	NM_001159637	0.56
		Nicotinate Phosphoribosyltransferase (Q8CC86)	*Naprt*	NM_172607	0.90

## Discussion

Brown adipocyte-mediated thermogenesis plays a key role in systemic energy homeostasis and age-related loss of brown adipocyte function may exacerbate the onset of metabolic diseases. Here we show that aging in brown adipose tissue results in changes of different metabolic pathway clusters, related mainly to energy, nucleotide and B-vitamin metabolism. These findings suggest that alterations of several polar intermediates involved in these pathways may act as a biomarker of age-related damage to brown adipocytes and could contribute to the age-dependent impairment of brown adipocyte function. Exploring this observation, we identify and annotate several intermediates in the context of the three different metabolic clusters whose concentrations are significantly correlated to the functional decline of brown adipocytes observed in aged mice. Investigation of circulating concentrations of polar metabolites in plasma samples of young and aged mice resulted in changes of molecular pathways with only limited overlap with the metabolites observed in aging BAT. While in general terms pathways linked to nucleotide and B vitamin metabolism were similarly affected in BAT and plasma, the overlap in energy metabolism-related pathways was not strongly evident on the levels of individual metabolites or enriched pathways. This discrepancy is likely due to the fact that circulating metabolites originate from multiple organ sources within the organism. This observation thus supports the notion that age-related effects indeed occur in BAT and are not likely due metabolite changes derived from the perfusing blood. A high relevance of these processes to brown adipocytes compared to other cells within BAT is further supported by the observation that expression of several metabolic enzymes linked to these pathway clusters was found to be enriched in brown adipocytes compared to the non-adipocyte SVF.

The integration of metabolomic, proteomic and transcriptional analyses revealed nucleotide metabolism as main pathway affected by age-dependent changes in BAT. Given the intracellular utilization of purine and pyrimidine nucleotides as substrate for nucleic acid synthesis, the relevance of these changes in nucleotide metabolite pools may be linked to a reduced brown adipogenesis capacity, which has been observed in aged BAT^10,12^. Consistent with this notion, a brown adipose tissue-specific decrease of precursors of purine synthesis, such as ADP-ribose and ribose-5´-phosphate, may result in an impaired replenishment of the purine nucleotide pool during aging. In order to prevent an eventual purine depletion, BAT may therefore increase the concentration of precursor purine species guanosine and inosine, which can be re-used by salvage pathway enzymes to synthetize purine nucleotides in a less energy-consuming process. Supporting this hypothesis, an increase of intracellular purine metabolism in active proliferative states, during the G1 and S phases of the cell cycle has been demonstrated^28^ and significant increases of intracellular 5´-PRPP concentrations have been shown in actively proliferating tumor cells, suggesting the requirement of intermediates involved in de novo synthesis to maintain appropriate purine flux^29^. In our analysis, enzymes of de novo purine nucleotide synthesis are decreased in aged BAT, including ADP-sugar pyrophosphatase (or nudix hydrolase 5, *Nudt5*) and multifunctional protein ADE2 (*Paics*), the latter taking part in the assembly of the purinosome complex. Interaction of purine metabolism with brown adipocyte energy homeostasis has been demonstrated via co-localization of a substantial fraction of the purinosome complex in proximity to mitochondria, thereby allowing efficient purine nucleotides de novo synthesis^30^. In this context, mitochondrial oxidative stress has been shown to contribute to cell-autonomous dysfunction during BAT-aging, characterized by excessive formation of reactive oxygen species (ROS) which in turn cause oxidative damage to DNA, cellular lipids and proteins^31–33^. Moreover, changes of salvageable purine concentrations, e.g. inosine and adenosine, have been found in different human cells during aging, indicating an age-dependent enhancement of cellular efflux of these intermediates^34^, and analysis of circulating metabolites in human individuals at different ages revealed a positive correlation with aging for some purine intermediates, including hypoxanthine, xanthine and uric acid^35–37^. Among the signaling pathways regulating purine synthesis, nutrient-sensing mammalian target of rapamycin (mTOR) has been proposed. mTORC1 enhances de novo purine biosynthesis and its activity is regulated by intracellular purine nucleotide pools, partly due to alterations in the level of activated GTP-bound Rheb signaling protein^38^. In addition, several lines of evidence indicate that purine catabolism in adipose tissue could be enhanced during metabolic disorders. Hyper-secretion of uric acid, a product of purine nucleotide breakdown, was found to be associated with obesity, visceral fat accumulation and type 2 diabetes in humans^39,40^. However, hyperuricemia has also been reported in obese mice, due to an enhancement of xanthine oxidoreductase activity in white adipose tissue^41^. Concerning pyrimidine metabolites, their role in brown and white adipose tissue metabolism during aging remains to be resolved. A previous study reported increased concentrations of nucleotides cytidine-5′-monophosphate (CMP) and uridine monophosphate (UMP) in WAT from high-fat diet fed mice, suggesting a functional relevance for pyrimidine intermediates during diet-dependent remodeling of adipose tissue^42^. These findings suggest that adipose tissue exerts an active role in nucleotide catabolism and alterations in the amounts of purine and pyrimidine intermediates may impair physiological adipose tissue function.

Our proteomic data analysis indicates depletion of enzymes involved in glucose oxidation processes, e.g. pyruvate metabolism and TCA cycle, suggesting that changes of metabolic intermediates involved in pyruvate metabolism may be due to reduced expression of these proteins. Specifically, age-related enrichment of two glycolytic enzymes was detected, hexokinase 1 and phosphoglycerate mutase 2, suggesting a potential increase of glucose breakdown in BAT of aged animals. Given that hexokinase 1 catalyzes the first, rate-limiting step of glycolysis, an increase of this protein during BAT-aging may be physiologically relevant, causing an age-dependent shift of the metabolite flux away from full mitochondrial oxidation towards increased glycolysis. However, the age-related decline in phosphoenolpyruvate levels could also indicate a less efficient metabolite flux and thus a general impairment of nutrient metabolism in aged BAT.

Lastly, our analysis introduced changes in histamine as a biomarker of mast cells in BAT. The role of this cell type in brown adipogenesis remains only partially understood, and reports have associated mast cells with beneficial as well as detrimental effects on brown adipocyte formation and thermogenesis. An early report demonstrated that mast cell ablation or stabilization, i.e. the pharmacological prevention of secretory release of the mast cells’ granular components, results in improved metabolic health, reduced inflammation of WAT and increased expression of UCP1 in BAT, among other improved metabolic phenotypes^25^. Similarly, inactivation of mast cells may induce browning of murine iWAT^24^. However, another study has previously linked mast cell-derived histamine and hypothalamic regulation of thermogenesis, although the underlying mechanism remains not fully elucidated^43^. Lastly, a more recent study showed that mast cells in human WAT may enhance the cold-induced browning process, suggesting that this cell type may act distinctively under specific physiological conditions^44^. The findings reported here suggest that secreted factors from mast cells may exert negative effects on brown adipocyte formation and correlates with aging. It remains to be determined whether a distinct physiological context, such as adrenergic activation, may further modify the role of mast cells in brown adipogenesis and thermogenesis.

In summary, we here show that aging results in changes of different polar metabolic clusters in BAT, including intermediates related to energy, nucleotide and B-vitamin metabolism. In particular, we detected prominent age-related alterations in the nucleotide metabolism cluster, resulting in reduced concentrations of purine intermediates of de novo synthesis and increased levels of salvageable purines. Therefore, metabolites and genes responsible for purine metabolism may serve as biomarkers to further define the age-dependent decline of brown adipocytes and their thermogenic function.

## Methods

### Animal experiments

All procedures were approved by the ethics committee for animal welfare of the State Office of Environment, Health, and Consumer Protection (State of Brandenburg, Germany) and all methods were performed in accordance with the relevant guidelines and regulations. The experiments are reported in accordance with ARRIVE guidelines whenever applicable. Blinded analyses were performed whenever feasible but animals’ age is clearly evident during handling and also the phenotypic changes in aged BAT potentially allow for recognition of animals’ age during histological analyses. Male C57BL/6-J mice (Charles River Laboratories, Sulzfeld, Germany) were housed in a controlled environment (22 °C ± 2 °C, 12/12 h light/dark cycle) and maintained on a standard diet (Ssniff, Soest, Germany). Cold exposure was conducted as described before^14^. In brief, to protect aged animals from high stress due to rapid temperature reduction, room temperature was reduced stepwise every other day, to 15 °C (day 0) and 10 °C (day 2), until reaching 5 °C on the fourth day of exposure, at which animals were kept for an additional three days before organ collection. Animals were killed by cervical dislocation. Adipose tissue depots were isolated, immediately frozen in liquid nitrogen, and stored at − 80 °C until further analysis. Tissues were ground while kept frozen in liquid nitrogen and sample powder was aliquoted for further analyses.

### Stromal vascular fraction and adipocytes isolation

SVF and mature adipocytes were isolated from interscapular brown adipose tissue and inguinal white adipose tissue of male mice aged 8 weeks. Freshly collected tissue was minced and subsequently digested in a sterile solution of 2 mg/ml of type II Collagenase (CellSystems GmbH, Troisdorf, Germany) in HBSS medium (PAN-Biotech GmbH, Aidenbach, Germany) containing 3.5% bovine serum albumin (BSA; Carl Roth GmbH, Karlsruhe, Germany). Samples were digested for 1 h at 37 °C with gentle agitation. Fetal bovine serum (PAN-Biotech GmbH, Aidenbach, Germany) was then added to the digestion solution to reach approximately 10% of the total volume; the digest was centrifuged at 4 °C for 10 min. Supernatants, containing the floating adipocyte fraction, were removed and SVF-pellets were resuspended in HBSS medium containing 2% FBS and filtered through 100 µm cell strainers (Fisher Scientific, Schwerte, Germany) to remove undigested tissue fragments. A further centrifugation step at 4 °C was performed, and cells were resuspended in sterile erythrocyte lysis buffer containing 0.15 M Ammonium Chloride, 0.01 M Potassium bicarbonate and Na-EDTA (Sigma-Aldrich, Darmstadt, Germany) and incubated on ice for 3 min. The solution was then filtered through a 40 µm cell strainer (Fisher Scientific, Schwerte, Germany) and centrifuged at 4 °C for 5 min. The resulting pellet was utilized for RNA extraction and gene expression analysis. For mature brown and white adipocyte isolation, the supernatant from the first centrifugation step after digestion was filtered through a 150 µm mesh and washed with an equal volume of pre-warmed (37 °C) Dulbecco´s Modified Eagle Medium (D-MEM; PAN-Biotech GmbH, Aidenbach, Germany) containing 10% FBS (washing media). The solution was centrifuged at low speed (200 rpm) to allow adipocytes to float as layer on top of the solution. The top layer was then transferred into a clean collection tube and fresh washing media was added. Centrifugation and washing steps were repeated until the solution underneath the adipocyte layer was clear. Adipocytes from the supernatant were gathered while ensuring only minimal washing solution was collected and were stored frozen at − 80 °C until further processing for gene expression analysis.

### Quantitative real-time PCR

RNA was isolated from ground nitrogen-frozen tissue powder, SVF or mature adipocytes by using an RNA MiniPrep Kit (Zymo Research, Freiburg, Germany). Purified RNA was reversely transcribed into cDNA using a high capacity cDNA reverse transcription kit (Thermo Fisher Scientific, Dreieich, Germany). Quantitative real-time PCR quantification was performed using Maxima SYBR Green/ROX qPCR Master Mix (Thermo Fisher Scientific, Dreieich, Germany) and the Real-Time PCR system CFX384 Touch (Bio-Rad, München, Germany). To amplify specific target genes, intron-spanning primers were used (Supplementary Table S5).

### Metabolomic analysis

Comparative metabolomic analysis was performed by MetaSysX GmbH (Potsdam-Golm, Germany) on interscapular brown adipose tissue samples from C57BL/6-J male mice aged 2.5, 5, 10, 15, 21 and 25 months as described before^14^. Metabolomic analysis of plasma was performed on samples collected from 8- and 65-weeks-old mice. The lipophilic extraction phase was used for LC–MS lipidomic analysis, the hydrophilic extraction phase was split to perform LC–MS and GC–MS metabolomic analysis, according to the protocol described previously^14^. GMP, inosine, L-glutamine, UMP, cytidine, thiamine pyrophosphate, riboflavin, pyridoxamine, nicotinamide riboside, acetyl phosphate, glutathione and 5-hydroxy–lysine were identified in the frame of the mass spectrometer from the [M + H] + precursor in positive ionization mode; ribose-5´-phosphate, ADP-ribose, guanosine, deoxyguanosine, adenosine, UMP-N-acetyl glucosamine, phosphoenolpyruvic acid and D-glucuronic acid were identified in the frame of the mass spectrometer from the [M –H] – precursor in negative ionization mode. Non-classical polar metabolites, decenedioic acid and geraniol, derived from the [M + NH4] + positive precursor, whereas 2-hydroxy-decanedioic acid derived from [M + OAc] – precursor. Pathway analysis of identified polar metabolites was performed using the software MetaboAnalyst (https://www.metaboanalyst.ca/home.xhtml) by selecting the hypergeometric test algorithm for the over-representation analysis^45,46^. The Mouse KEGG database as reference metabolomic library was selected for the analysis^47–49^.

### Analysis of brown adipose tissue proteome

Proteomic analysis was performed in young and old animals by conducting two independent comparisons as described before^14^. In brief, following collection of BAT extracts from five age-matched mice, each sample was generated by pooling equal amounts of tissue powder. Each sample pair was measured in duplicate and either the young or the aged sample of each pool were labeled with 18O prior to analysis. Proteins were separated by 12% polyacrylamide gel electrophoresis, and equally sized pieces of Coomassie stained protein bands were removed from all lanes of the gel. Protein digestion and in-gel 16O/18O-labeling were performed as described^14^. Subsequently, mass spectrometric analysis was performed using Nano-LC–ESI–MS/MS (Orbitrap Elite, Thermo Fisher Scientific; Schwerte, Germany). The MS/MS spectra were processed using MASCOT server (version 2.2, Matrix Science Ltd., London) and the annotated proteins were searched in UniProtKB/Swiss-Prot database MOUSE 2013okt (51,193 sequences; 24,573,350 residues). Quantification was performed using the Mascot Distiller Quantitation Toolbox (version 2.2.1.2, Matrix Science Ltd., London; Great Britain) and was based on calculations of isotope intensity ratios of at least two tryptic peptides with individual MASCOT scores indicating at least homology. Relative heavy/light protein ratios (H/L) were calculated from the intensity-weighted average of all peptide ratios. Age-dependent regulation of proteins in aged BAT-samples were determined based on H/L ratio < 1 for down-regulated proteins and on H/L ratio > 2.5 for up-regulated proteins.

### Histological analysis

BAT was fixed overnight in 4% formalin at room temperature, then dehydrated, paraffin embedded, and serially sectioned into slices of 5 μm thickness and placed onto frosted microscope glass slides. After rehydration, mast cell granules were stained with 0.1% (w/v) aqueous Toluidine blue O (Merck Millipore, Darmstadt, Germany) for 3 min, quickly dehydrated and mounted with Flouromount G (Thermo Fisher Scientific, Dreieich, Germany). For immunofluorescence detection of mast cells, heat-mediated antigen retrieval was conducted by placing rehydrated slices in blocking buffer (21.3 mM Citric acid, 1.5% hydrogen peroxide (v/v) and 1% SDS (w/v) in ddH2O) for 15 min at room temperature, followed by second blocking buffer incubation (40 mM Tris and 1.2 mM EDTA in ddH2O) in a microwave for 5 min at 400 W. Slides were washed and permeabilized in PBS, 0.4% Triton X-100 (v/v) for 10 min at room temperature. After blocking with 3% BSA/PBS for 60 min at RT, sections were incubated with an anti-Carboxypeptidase A3 primary antibody (Antibodies-online GmbH, Aachen, Germany), overnight in a humidified chamber at 4 °C. Sections were washed and incubated for 60 min with a secondary antibody conjugated with Alexa Fluor 488 (Abcam, Cambrige, United Kingdom) and DAPI nucleus staining (BioLegend, San Diego, CA, USA) at room temperature in the dark. Autofluorescence was reduced by incubation with 0.3% Sudan Black B (Sigma Aldrich, Taufkirchen, Germany) in 70% ethanol for 20 min, then sections were washed and mounted with Fluoromount G (Thermo Fisher Scientific, Dreieich, Germany). Slices were photographed at 600-fold magnification and analyzed with Image J software (NIH, Bethesda, MA, USA)^50^. Ratios were calculated as the number of Toluidine blue O or CPA3 positive cells over total tissue area (mm^2^). For Toluidine blue O staining, 1–2 sections from 8 animals per age group were taken; for CPA3 immunofluorescence, 1–2 sections from 7 animals per age group were analyzed.

### Isolation and differentiation of pre-adipocytes

Primary pre-adipocytes were isolated using flow cytometry and subsequently cultivated and differentiated as described before^14,51^. In brief, the adipose tissue stromal vascular fraction was isolated as described above, and antibody staining to label specific cell surface markers was as follows: Anti-mouse stem cell antigen-1 (Sca1; clone D7; isotype Rat IgG2a; 0.5 μg/mL) for positive selection. Anti-mouse antibodies against cluster of differentiation 31 (CD31; clone 390; isotype Rat IgG2a; 1 μg/mL) and anti-mouse CD45 (clone 30-F11; isotype Rat IgG2b; 2.5 μg/mL), directed against endothelial and hematopoietic cells, respectively, were used for negative selection. Cell were plated and expanded for approximately one week before cells were seeded at defined densities for experimental setup. On the day before start of differentiation, co-cultures were seeded by combining pre-adipocytes with the indicated percentual proportions of cells of the mastocytoma cell line p815 ^26^. Differentiation was conducted in the presence of a standard cocktail of growth medium supplemented with 5 μg/mL human insulin, 50 μM indomethacin, 1 μM dexamethasone, 0.5 μM isobutylmethylxanthine (IBMX), 1 nM T3 for 48 h. Medium was then changed to contain 5 μg/mL human insulin, 1 nM T3 for the remainder of the differentiation course. Adipogenesis was conducted for 10 days, cells were then harvested for mRNA-isolation and gene expression analysis was performed as described before^14^. For conditioned media-exposure, the brown pre-adipocyte cell line, WT1, was differentiated for a total of 10 days as described before^14,52^. At day 9 of differentiation, culture media was exchanged for conditioned media from 24-h cultures of p815 mast cells and were compared to control culture media that was conditioned by cultivation of undifferentiated WT1 pre-adipocytes for 24 h.

### Statistical analyses

Statistical analysis was conducted using GraphPad Prism software. Outliers were tested and removed after using the ROUT method. Significance levels of differences between groups were evaluated using either an unpaired two-tailed Student’s t-test or non-parametric Mann–Whitney-U test when comparing the means of two groups, or non-parametric Kruskal–Wallis tests for multi-group comparisons of more than two groups.

## Supplementary Information

Supplementary Information.
