# Supplementary material for: Identification of biomarkers of brown adipose tissue aging highlights the role of dysfunctional energy and nucleotide metabolism pathways
Source: Sci Rep. 2021 Oct 7;11:19928. doi: 10.1038/s41598-021-99362-1 (PMC8497523; doi:10.1038/s41598-021-99362-1)
Supplement: Supplementary file 1 — Supplementary Information. [file 41598_2021_99362_MOESM1_ESM.pdf]

Identification of biomarkers of brown adipose tissue aging highlights the role of dysfunctional energy and nucleotide metabolism pathways

Carola Mancini[1,2], Sabrina Gohlke[1], Francisco Garcia-Carrizo[1], Vyacheslav Zagoriy[3], Heike Stephanowitz[4], Tim J. Schulz[1,2,5*]

[1]Department of Adipocyte Development and Nutrition, German Institute of Human Nutrition Potsdam-Rehbrücke, Nuthetal, Germany

[2]German Center for Diabetes Research (DZD), München-Neuherberg, Germany

[3]metaSysX GmbH, Potsdam-Golm, Germany

[4]Leibniz-Forschungsinstitut für Molekulare Pharmakologie (FMP), Berlin, Germany

[5]Institute of Nutritional Science, University of Potsdam, Potsdam-Rehbrücke, Nuthetal, Germany

*Correspondence: Tim J. Schulz; German Institute of Human Nutrition, 114-116 Arthur-Scheunert-Allee, D-14558 Nuthetal, Germany, Phone: +49-33200 88 2110; Email: tim.schulz@dife.de

Supplementary Figure S1

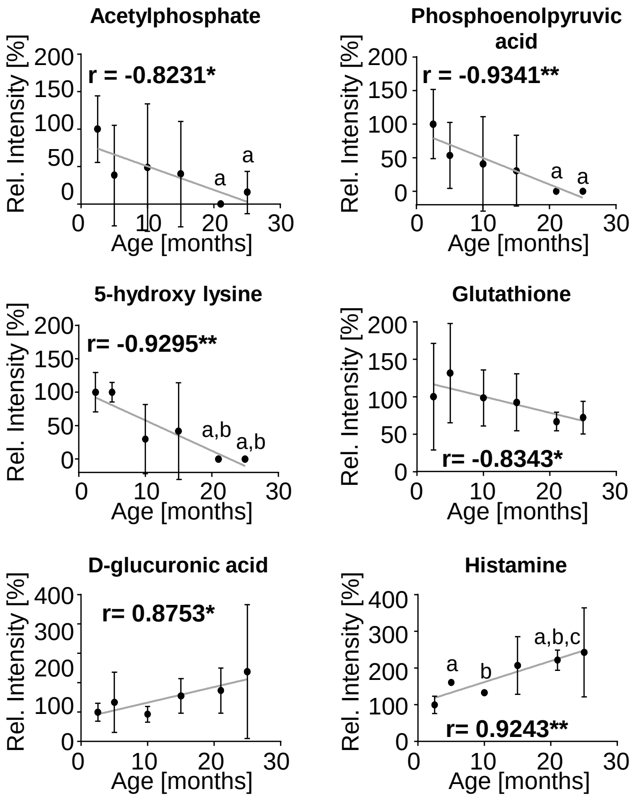

Supplementary Figure S2

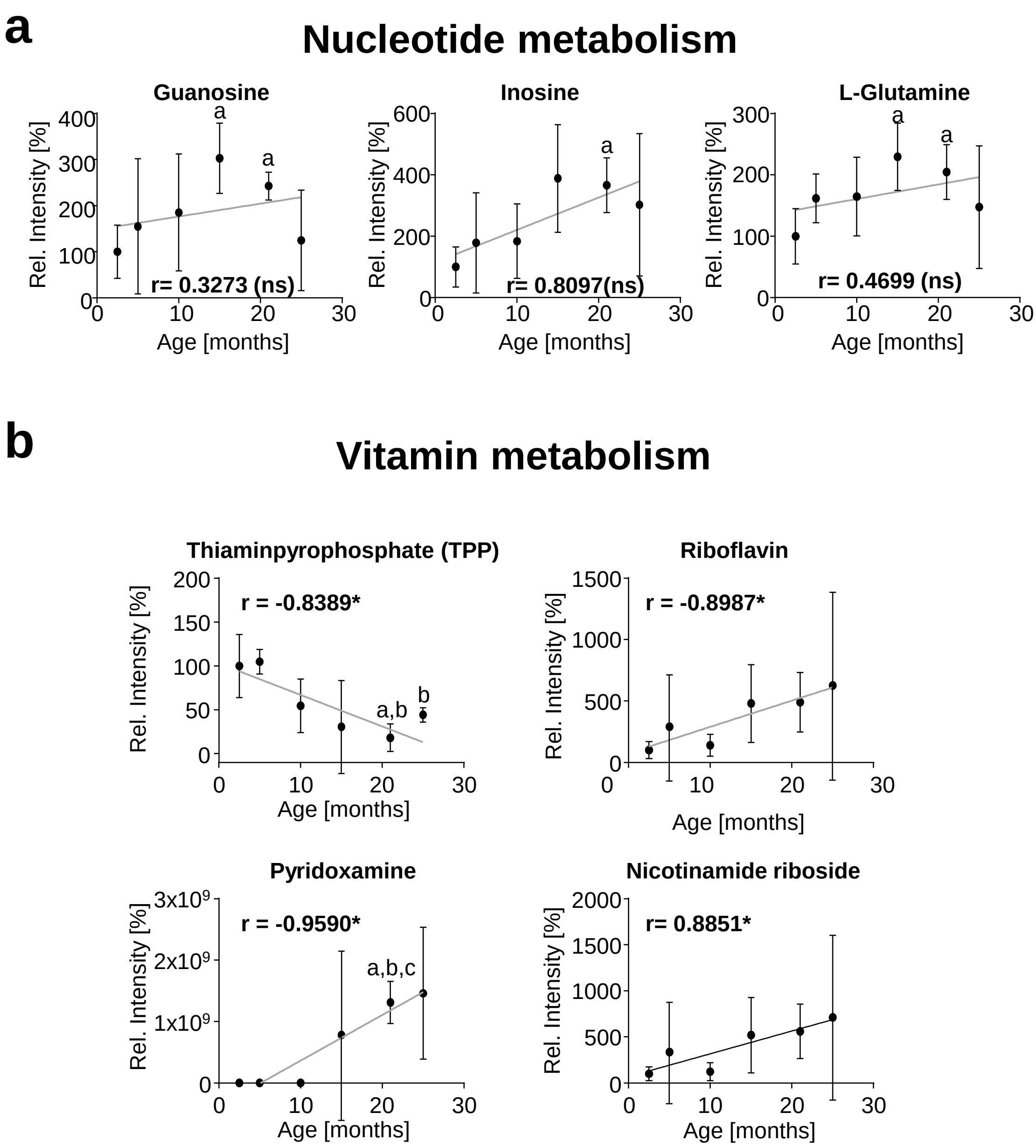

Supplementary Figure S3

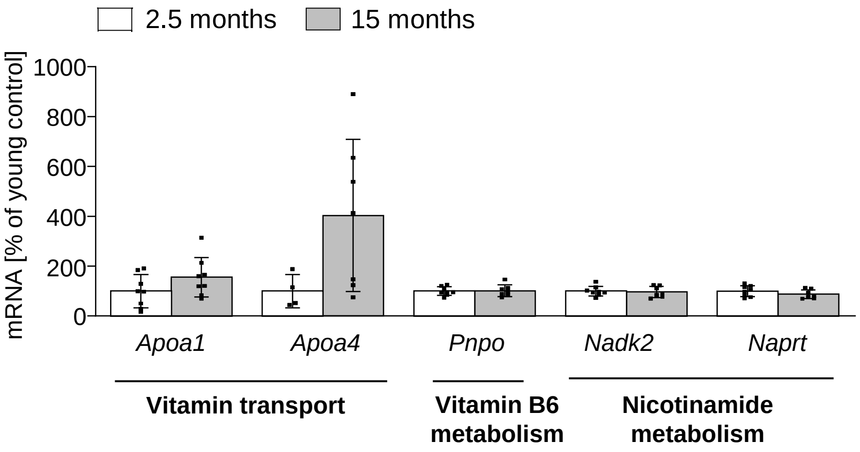

Supplementary Figure S4

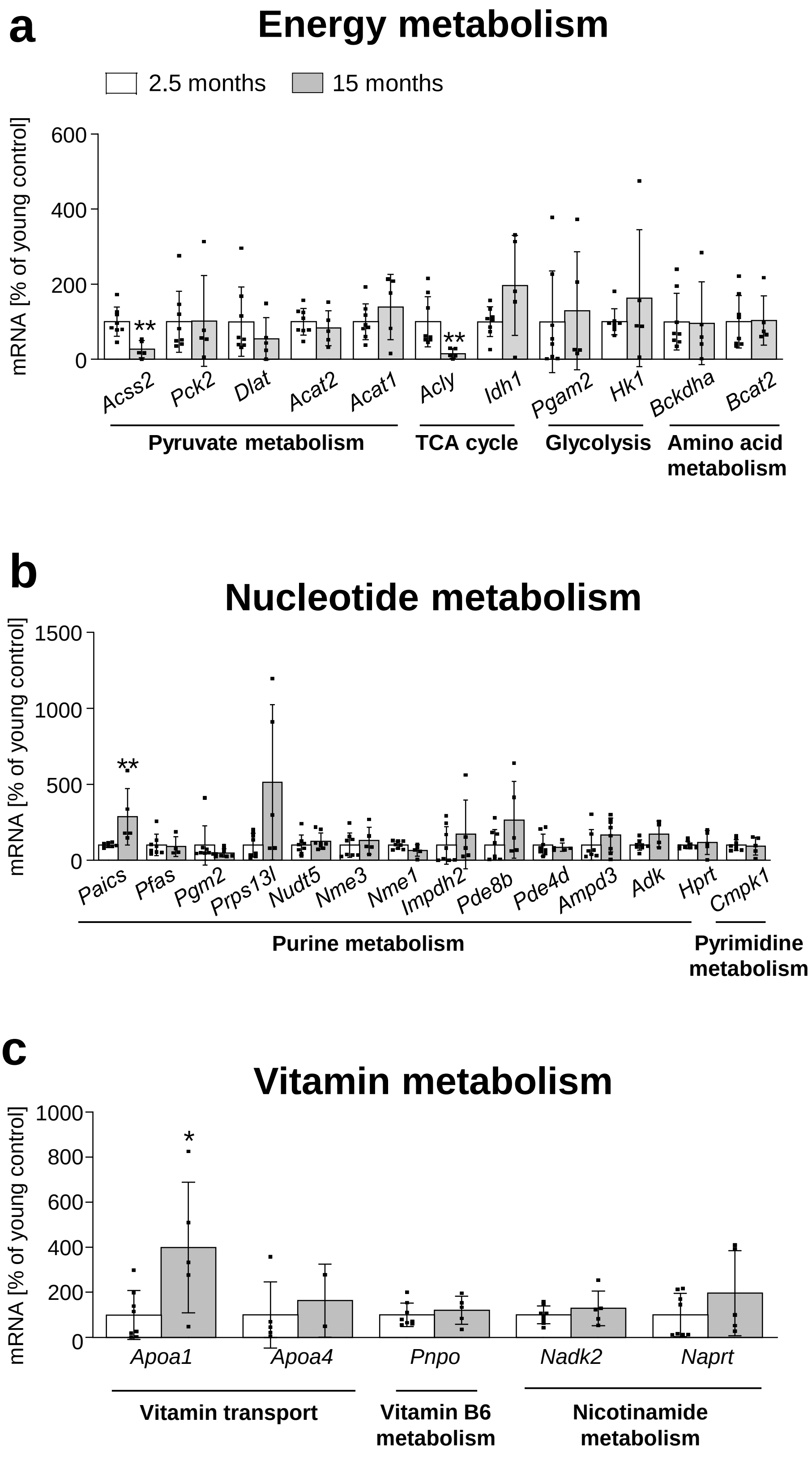

Supplementary Figure S5

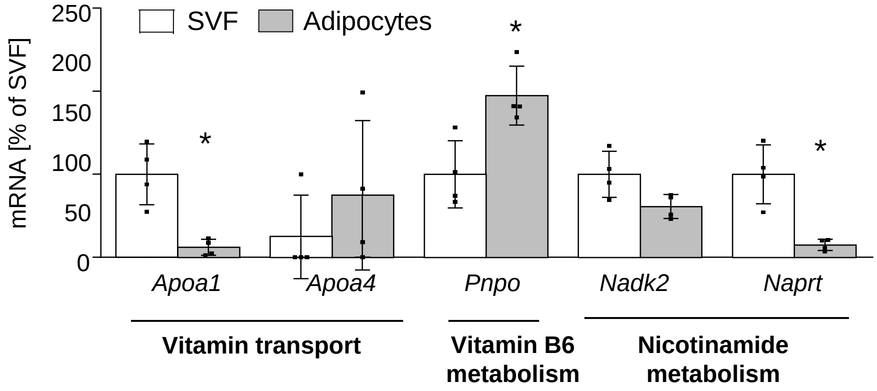

Supplementary Figure S6

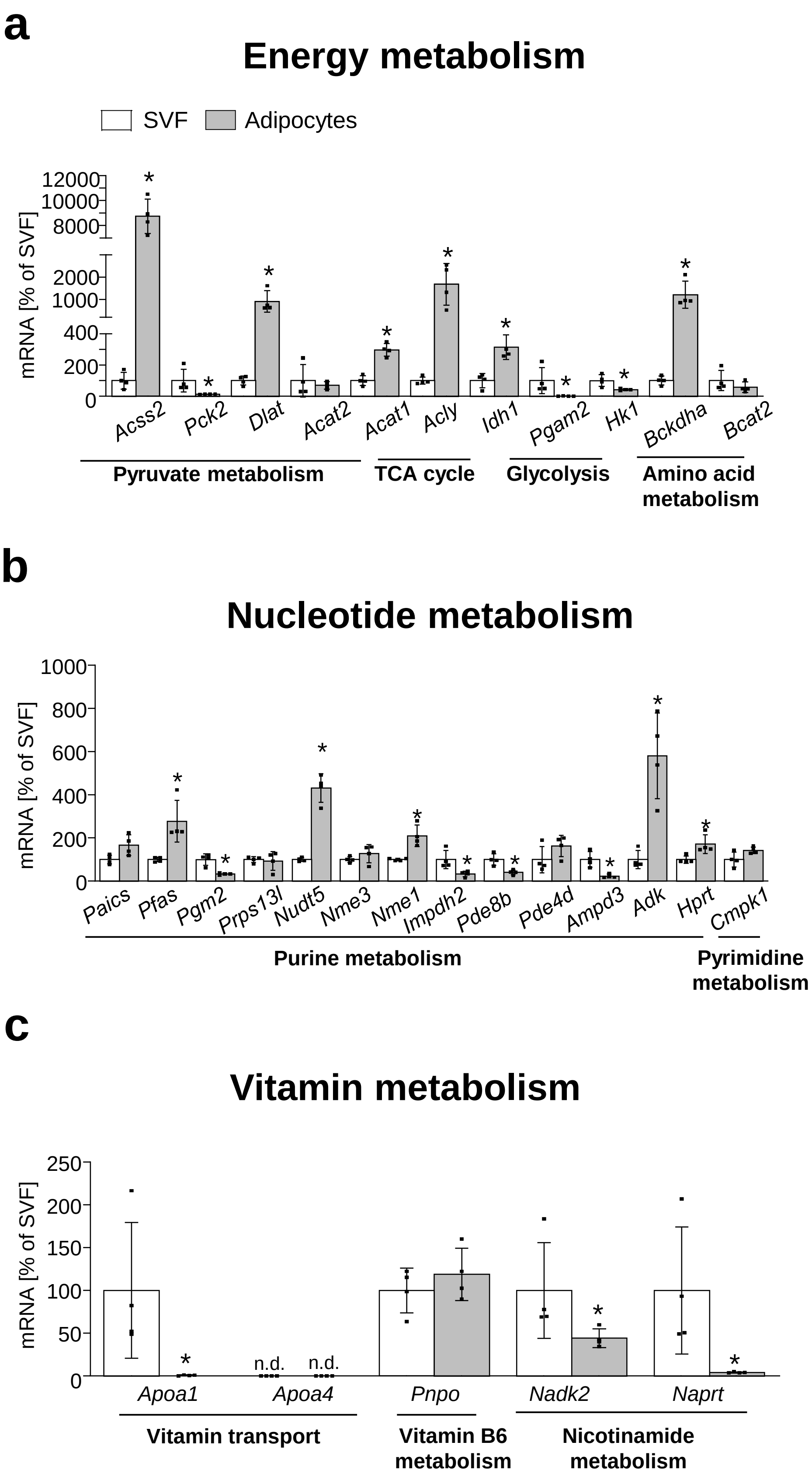

Supplementary Figure S7

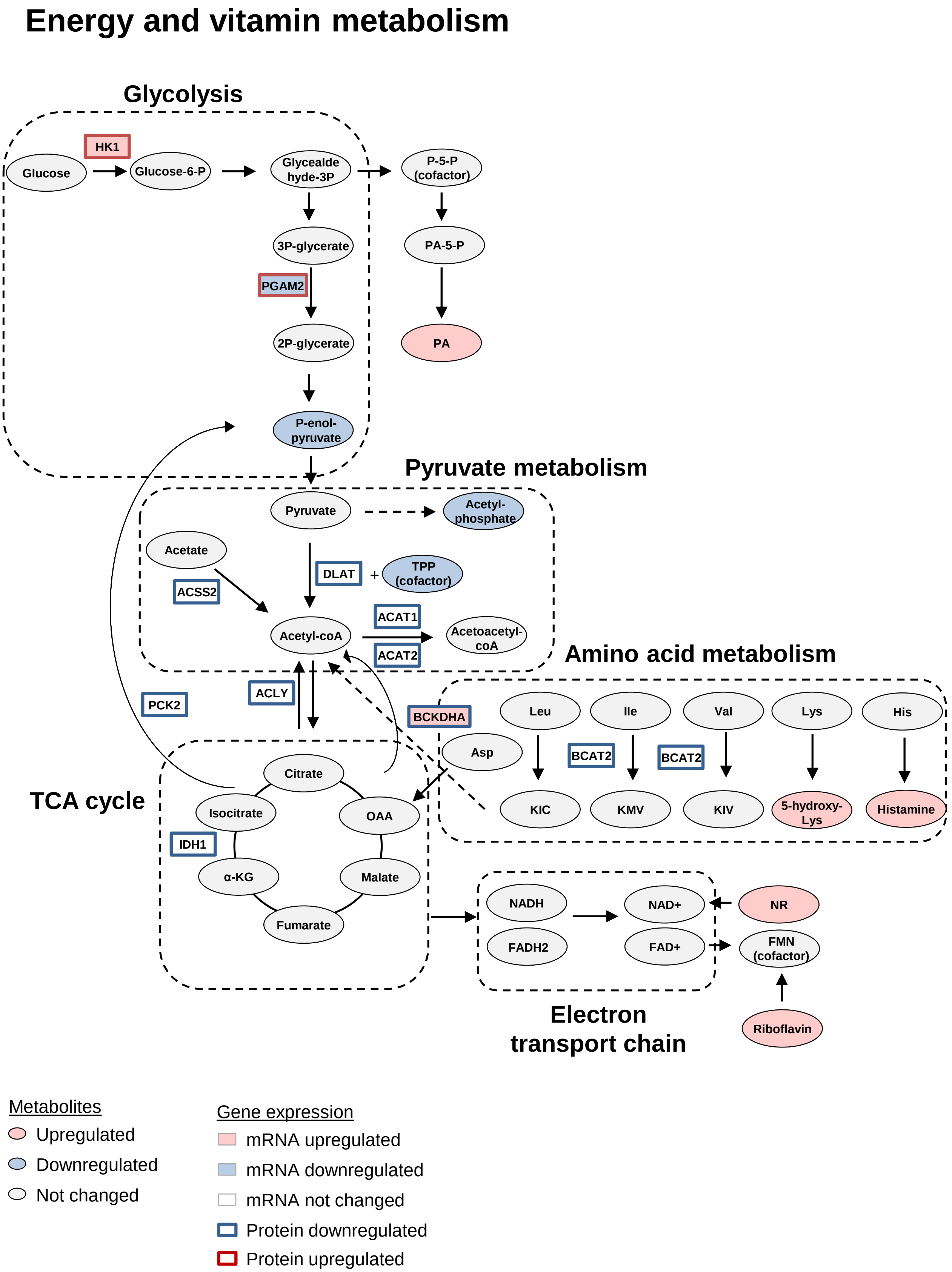

Supplementary Figure legends

Supplementary Figure S1. Spearman correlations of individual polar metabolites linked to energy metabolism that are significantly associated with aging in BAT. All metabolites were detected by LC-MS and GC-MS metabolomic analysis. Correlation coefficients (r) for individual metabolites were calculated to visualize age-dependent correlations of metabolic species. Individual peak intensities of all age groups were normalized to intensities measured in the 2.5 months control group. All data are depicted as mean ± SD (n = 3); *p <0.05 indicating significant correlation across all age groups; [a, b, c] p <0.05 comparing relative intensity of the indicated age group versus (a) 2.5 months, (b) 5 months or (c) 10 months, respectively, assessed by two-tailed unpaired t-test.

Supplementary Figure S2. (**a**) Spearman correlations of individual polar metabolites linked to nucleotide metabolism that are not significantly associated with aging in BAT but show statistically significant differences between individual age-groups. (**b**) Spearman correlations of individual polar metabolites linked to B-vitamin metabolism that are significantly associated with aging in BAT. All metabolites were detected by LC-MS and GC-MS metabolomic analysis. Correlation coefficients (r) for individual metabolites were calculated to visualize age-dependent correlations of metabolic species. Individual peak intensities of all age groups were normalized to intensities measured in the 2.5 months control group. All data are depicted as mean ± SD (n = 3); *p <0.05 indicating significant correlation across all age groups; [a, b, c] p <0.05 comparing relative intensity of the indicated age group versus (a) 2.5 months, (b) 5 months or (c) 10 months, respectively, assessed by two-tailed unpaired t-test.

Supplementary Figure S3. mRNA levels of B-vitamin metabolism genes comparing BAT samples of young (2.5 months; n = 8; white bars) and old (15 months; n = 7; grey bars) mice. Genes (see Suppl. Table S5 for full gene names) are organized according to pathway enrichment in proteome analysis comparing the similar age groups (Table 1). Data are expressed as percentage of young (2.5 months) control. Data are shown as mean ± SD, statistical differences assessed by non-parametric Mann-Whitney test.

Supplementary Figure S4. mRNA levels of genes regulating nutrient/ energy metabolism (**a**), nucleotide metabolism (**b**) and vitamin metabolism (**c**) comparing iWAT samples of young (2.5 months; n = 8; white bars) to aged (15 months; n = 5; grey bars) mice. Genes (see Suppl. Table S5 for full gene names) are organized according to pathway enrichment in proteome analysis comparing the similar age groups (Table 1). Data are expressed as percentage of young (2.5 months) control. Data are shown as mean ± SD; *p <0.05; **p <0.01, using non-parametric Mann-Whitney test.

Supplementary Figure S5. mRNA levels of B-vitamin metabolism genes assessed in SVF (white bars) and mature adipocytes (grey bars) isolated from BAT of 2.5-month old mice. Genes (see Suppl. Table S5 for full gene names) are organized according to pathway enrichment in proteome analysis comparing the similar age groups (Table 1). For each gene, data are expressed as percentage of BAT-derived SVF. Data are shown as mean ± SD (n = 4 in both groups); *p <0.05 assessed by non-parametric Mann-Whitney test.

Supplementary Figure S6. mRNA levels of genes regulating nutrient/ energy metabolism (**a**), nucleotide metabolism (**b**) and B-vitamin metabolism (**c**) assessed in SVF (white bars) and mature adipocytes (grey bars) isolated from iWAT of 2.5-months old mice. Genes (see Suppl. Table S5 for full gene names) are organized according to pathway enrichment in proteome analysis comparing the similar age groups (Table 1). For each gene, data are expressed as percentage of iWAT-derived SVF. Data are shown as mean ± SD (n = 4); *p <0.05 assessed by non-parametric Mann-Whitney test.

Supplementary Figure S7. Metabolic network recapitulating age-dependent regulation of nutrient/ energy metabolism and of vitamin metabolism observed in metabolomic, proteomic and transcriptional data analyses. Dashed lines depict different metabolic branches of energy metabolism: glycolysis, pyruvate metabolism, amino acid metabolism, TCA cycle and electron transport chain. Each oval represents a metabolite, while each box represents a gene/protein. As shown in the legend, color-coding is provided for metabolites, genes and proteins enriched (red) or depleted (blue) during BAT-aging. Metabolites are shown in gray when the concentration was not affected by aging in BAT. Color-coding of metabolites is based on the log2 fold change (FC) values calculated in comparison to the 2.5-month old control group (Supplementary Table S2). For each

protein and gene, age-dependent regulation is represented by the same color-coding; genes whose mRNA was not changed during BAT-aging are depicted in white boxes. Proteomic changes are based on heavy/light ratios comparing young vs aged mice (Table 1).

Supplementary Tables

Supplementary Table S1. Significantly regulated ($p < 0.05$) polar and non-polar metabolites identified by comparative metabolomic analysis (LC/GC-MS) performed on plasma samples from young (2 months) to aged (15 months) mice (n = 5 group). For each metabolite, log2 fold change (log2FC) was calculated comparing relative intensities of young vs aged mice. Plasma metabolites with age-related changes were grouped into functionally related sub-clusters: (1) nutrient/ energy metabolism, (2) nucleotide metabolism, (3) vitamin metabolism, (4) lipid metabolism and (5) xenobiotic metabolism. Statistical significance was assessed by two-tailed unpaired t-test between age groups; *p <0.05; **p < 0.01 vs young mice.

Cluster		Metabolite	Log2FC young vs. old
1	Energy metabolism	Citric acid	-0.50*
		Creatine	-0.39*
		Phosphocreatine	1.44*
		Fructose	-0.36*
		Glucose-1-phosphate	-0.39*
		Inositol	-0.77*
		Mannitol	-0.22*
		Mannose	-0.41*
		Fucose	0.80**
		Glyceraldehyde 3-phosphate	0.74*
		Glutamic acid	-0.76*
		L-Glutamine	0.63**
		Hydroxyproline	-1.31**
		Proline	0.46*
		Histidine	0.40*
		Ornithine	0.52*
		Trimethyllysine	-0.70**
		Betaine	0.47*
		Tyrosine	-0.24*
		Methylglutaric acid	-0.56*
		Spermidine	-0.82*
		Succinic acid	-0.33*
		Threonic acid	-1.70*
		3-Hydroxybutyric acid	-1,34**

2	**Nucleotide metabolism**	Acetylaspartic acid	-0.78*
		Acetylysine	-0.71*
		Acetyltryptophane	0.88*
		Allantoin	-0.30*
		Guanosine	-0.96**
		Hypoxanthine	-5.85**
		Xanthine	-3.82**
		Uric acid	-0,49*
		Uracile	-0.65*
		Uridine	-0.84*
		3-Ureidopropanoic acid	-1,40*
3	**Vitamin metabolism**	Thiamine	-1.49*
		4-Pyridoxic acid	-1,41*
4	**Lipid metabolism**	Choline	-0.43*
		Glycerol-3-phosphate	-0.50*
		Hydroxycapric acid	-2.28*
		Carnitine	0.54*
		Cysteinesulfinic acid	-2.68*
5	**Xenobiotic metabolism**	5-Methoxyindole-3-acetic acid	-1,37*
		Benzoic acid	-0.24*

Supplementary Table S2. Age-dependent regulation of nutrient/ energy metabolites in aging-BAT.

Table depicting each polar intermediate clustering in the energy/ nutrient metabolism group; log2 fold changes (log2FC) were calculated comparing single age-groups. In bold are depicted significant changes of log2FC. Statistical significance was assessed by two-tailed unpaired t-test between age-groups; [a, b, c] $p < 0.05$ vs BAT of 2.5-, 5-, 10-month old mice, respectively.

**Cluster 1 – Nutrient/energy metabolism**	**Log2FC**					
**Acetylphosphate**	**2.5 mo**	**5 mo**	**10 mo**	**15 mo**	**21 mo**	**25 mo**
**2.5 mo**	--	-1.38	-1.03	-1.31	**n.d.** (a)	**-2.66** (a)
**5 mo**	--	--	0.35	0.07	n.d.	-1.27
**10 mo**	--	--	--	-0.28	n.d.	-1.62
**15 mo**	--	--	--	--	--	-1.35
**21 mo**	--	--	--	--	--	--
**25 mo**	--	--	--	--	--	--
**Phosphoenolpyruvic acid**	**2.5 mo**	**5 mo**	**10 mo**	**15 mo**	**21 mo**	**25 mo**
**2.5 mo**	--	-0.90	-1.30	-1.71	**n.d.** (a)	**n.d.** (a)
**5 mo**	--	--	-0.39	-0.81	n.d	n.d.
**10 mo**	--	--	--	-0.42	n.d.	n.d.
**15 mo**	--	--	--	--	n.d.	n.d.
**21 mo**	--	--	--	--	--	n.d.
**25 mo**	--	--	--	--	--	--
**5-hydroxylysine**	**2.5 mo**	**5 mo**	**10 mo**	**15 mo**	**21 mo**	**25 mo**
**2.5 mo**	--	0.00	-1.74	-1.26	**n.d.** (a)	**n.d.** (a)
**5 mo**	--	--	-1.74	-1.26	**n.d.** (b)	**n.d.** (b)
**10 mo**	--	--	--	0.48	n.d.	n.d.

**15 mo**	--	--	--	--	n.d.	n.d.
**21 mo**	--	--	--	--	--	n.d.
**25 mo**	--	--	--	--	--	--
**Histamine**	**2.5 mo**	**5 mo**	**10 mo**	**15 mo**	**21 mo**	**25 mo**
**2.5 mo**	--	**0.69** (a)	0.41	1.05	**1.15** (a)	1.28
**5 mo**	--	--	**-0.27** (b)	0.36	0.46	0.59
**10 mo**	--	--	--	0.64	**0.74** (c)	0.87
**15 mo**	--	--	--	--	0.10	0.23
**21 mo**	--	--	--	--	--	0.13
**25 mo**	--	--	--	--	--	--
**Glutathione**	**2.5 mo**	**5 mo**	**10 mo**	**15 mo**	**21 mo**	**25 mo**
**2.5 mo**	--	0.40	-0.02	-0.11	-0.58	-0.47
**5 mo**	--	--	-0.42	-0.51	-0.97	-0.87
**10 mo**	--	--	--	-0.09	-0.56	-0.45
**15 mo**	--	--	--	--	-0.47	-0.36
**21 mo**	--	--	--	--	--	0.11
**25 mo**	--	--	--	--	--	--
**D-glucuronic acid**	**2.5 mo**	**5 mo**	**10 mo**	**15 mo**	**21 mo**	**25 mo**
**2.5 mo**	--	0.41	-0.11	0.63	0.79	1.25
**5 mo**	--	--	-0.52	0.22	0.38	0.84
**10 mo**	--	--	--	0.74	0.90	1.36
**15 mo**	--	--	--	--	0.16	0.61
**21 mo**	--	--	--	--	--	0.45
**25 mo**	--	--	--	--	--	--

Supplementary Table S3. Age-dependent changes of nucleotide metabolism in BAT.

Table representing age-dependent regulation of intermediates of nucleotide metabolism. Log2FC for each metabolite was calculated comparing single age-groups. In bold are depicted significant changes of log2FC. Statistical significance was assessed by two-tailed unpaired t-test between age-groups; [a, b, c, d] $p < 0.05$ vs BAT of 2.5-, 5-, 10- and 15-month old mice, respectively).

**Cluster 2 – Nucleotide metabolism**	**Log2FC**					
**GMP**	**2.5 mo**	**5 mo**	**10 mo**	**15 mo**	**21 mo**	**25 mo**
**2.5 mo**	--	-0.54	-0.41	-0.81	-0.92	-1.01
**5 mo**	--	--	0.12	-0.27	-0.38	-0.47
**10 mo**	--	--	--	-0.39	-0.50	-0.59
**15 mo**	--	--	--	--	-0.11	-0.19
**21 mo**	--	--	--	--	--	-0.08
**25 mo**	--	--	--	--	--	--
**Ribose-5´-phosphate**	**2.5 mo**	**5 mo**	**10 mo**	**15 mo**	**21 mo**	**25 mo**
**2.5 mo**	--	0.24	-0.05	-0.76	-1.14	-0.63
**5 mo**	--	--	-0.30	-1.00	-1.38	-0.87
**10 mo**	--	--	--	-0.70	-1.08	-0.57
**15 mo**	--	--	--	--	-0.38	0.13
**21 mo**	--	--	--	--	--	0.51
**25 mo**	--	--	--	--	--	--
**ADP-ribose**	**2.5 mo**	**5 mo**	**10 mo**	**15 mo**	**21 mo**	**25 mo**
**2.5 mo**	--	-0.63	-0.62	-0.51	-1.09	-1.40
**5 mo**	--	--	0.00	0.12	-0.46	-0.71
**10 mo**	--	--	--	0.11	**-0.46** (c)	**-0.77** (c)
**15 mo**	--	--	--	--	-0.58	-0.89
**21 mo**	--	--	--	--	--	-0.31
**25 mo**	--	--	--	--	--	--
**Deoxyguanosine**	**2.5 mo**	**5 mo**	**10 mo**	**15 mo**	**21 mo**	**25 mo**

**2.5 mo**	--	2.42	0.98	3.78	**3.61** (a)	3.88
**5 mo**	--	--	-1.43	1.35	1.18	1.45
**10 mo**	--	--	--	2.79	2.62	2.89
**15 mo**	--	--	--	--	-0.17	0.10
**21 mo**	--	--	--	--	--	0.27
**25 mo**	--	--	--	--	--	--
**Inosine**	**2.5 mo**	**5 mo**	**10 mo**	**15 mo**	**21 mo**	**25 mo**
**2.5 mo**	--	0.83	0.87	1.95	**1.97** (a)	1.59
**5 mo**	--	--	0.04	1.12	1.03	0.76
**10 mo**	--	--	--	1.07	0.99	0.71
**15 mo**	--	--	--	--	-0.08	-0.36
**21 mo**	--	--	--	--	--	-0.27
**25 mo**	--	--	--	--	--	--
**Guanosine**	**2.5 mo**	**5 mo**	**10 mo**	**15 mo**	**21 mo**	**25 mo**
**2.5 mo**	--	0.63	0.88	**1.59** (a)	**1.27** (a)	0.90
**5 mo**	--	--	0.25	0.96	0.64	0.27
**10 mo**	--	--	--	0.71	0.39	0.01
**15 mo**	--	--	--	--	-0.31	-0.69
**21 mo**	--	--	--	--	--	-0.37
**25 mo**	--	--	--	--	--	--
**L-glutamine**	**2.5 mo**	**5 mo**	**10 mo**	**15 mo**	**21 mo**	**25 mo**
**2.5 mo**	--	0.69	0.71	**1.19** (a)	**1.03** (a)	0.55
**5 mo**	--	--	0.02	0.50	0.34	-0.13
**10 mo**	--	--	--	0.48	0.31	-0.15
**15 mo**	--	--	--	--	-0.16	-0.63
**21 mo**	--	--	--	--	--	-0.47
**25 mo**	--	--	--	--	--	--
**UDP-N-acetylglucosamine**	**2.5 mo**	**5 mo**	**10 mo**	**15 mo**	**21 mo**	**25 mo**
**2.5 mo**	--	-0.15	-0.24	-0.20	-0.65	-0.87
**5 mo**	--	--	-0.09	-0.04	-0.50	-0.72
**10 mo**	--	--	--	0.04	-0.41	-0.63
**15 mo**	--	--	--	--	**-0.45** (d)	-0.67
**21 mo**	--	--	--	--	--	-0.22
**25 mo**	--	--	--	--	--	--
**UMP**	**2.5 mo**	**5 mo**	**10 mo**	**15 mo**	**21 mo**	**25 mo**
**2.5 mo**	--	-0.44	-0.30	-0.20	-0.39	-0.78
**5 mo**	--	--	0.14	0.05	-0.26	-0.34
**10 mo**	--	--	--	-0.09	-0.40	-0.48
**15 mo**	--	--	--	--	-0.31	-0.39
**21 mo**	--	--	--	--	--	-0.08
**25 mo**	--	--	--	--	--	--
**Cytidine**	**2.5 mo**	**5 mo**	**10 mo**	**15 mo**	**21 mo**	**25 mo**
**2.5 mo**	--	-0.28	-0.55	0.64	0.94	1.00
**5 mo**	--	--	-0.27	0.92	1.22	1.28
**10 mo**	--	--	--	1.19	1.49	1.55
**15 mo**	--	--	--	--	0.30	0.36
**21 mo**	--	--	--	--	--	0.06
**25 mo**	--	--	--	--	--	--

Supplementary Table S4. Age-dependent regulation of vitamin metabolism in BAT.

Table representing age-dependent changes of intermediates related to vitamin metabolism. Log2FC value for each metabolite was calculated comparing single age-groups, and significant changes are depicted in bold. Statistical significance was assessed by two-tailed unpaired t-test between age-groups; [a, b, c] $p < 0.05$ vs BAT of 2.5-, 5- and 10-month old mice, respectively.

**Cluster 3 – Vitamin B metabolism**	**Log2FC**					
**Thiaminpyrophosphate**	**2.5 mo**	**5 mo**	**10 mo**	**15 mo**	**21 mo**	**25 mo**
**2.5 mo**	--	0.07	-0.88	-1.72	**-2.47** [(a)]	-1.18
**5 mo**	--	--	-0.95	-1.78	**-2.54** [(b)]	**-1.25** [(b)]
**10 mo**	--	--	--	-0.84	-1.59	-0.30
**15 mo**	--	--	--	--	-0.75	0.54
**21 mo**	--	--	--	--	--	1.29
**25 mo**	--	--	--	--	--	--
**Riboflavin**	**2.5 mo**	**5 mo**	**10 mo**	**15 mo**	**21 mo**	**25 mo**
**2.5 mo**	--	1.53	0.48	2.26	2.29	2.65
**5 mo**	--	--	-1.05	0.73	0.76	1.12
**10 mo**	--	--	--	1.78	1.82	2.17
**15 mo**	--	--	--	--	0.03	0.39
**21 mo**	--	--	--	--	--	0.36
**25 mo**	--	--	--	--	--	--
**Pyridoxamine**	**2.5 mo**	**5 mo**	**10 mo**	**15 mo**	**21 mo**	**25 mo**
**2.5 mo**	--	n.d.	n.d.	22.90	**23.65** [(a)]	23.80
**5 mo**	--	--	n.d.	n.d.	**n.d.** [(b)]	n.d.
**10 mo**	--	--	--	n.d.	**n.d.** [(c)]	n.d.
**15 mo**	--	--	--	--	0.74	0.90
**21 mo**	--	--	--	--	--	0.15
**25 mo**	--	--	--	--	--	--
**Nicotinamide riboside**	**2.5 mo**	**5 mo**	**10 mo**	**15 mo**	**21 mo**	**25 mo**
**2.5 mo**	--	1.75	0.29	2.37	2.49	2.83
**5 mo**	--	--	-1.46	0.62	0.74	1.08
**10 mo**	--	--	--	2.08	2.20	2.54
**15 mo**	--	--	--	--	0.11	0.46
**21 mo**	--	--	--	--	--	0.35
**25 mo**	--	--	--	--	--	--

Supplementary Table S5: Primer sequences for quantitative real-time PCR.

Gene	Gene name	Sequence (5´to 3´)
Acat1	Acetyl-Coenzyme A acetyltransferase 1 (mitochondrial)	Fw: CTCAGCCGGCCACTAAACTT<br>Rev: GAAATAGGTAAACCTGCGCCC
Acat2	Acetyl-Coenzyme A acetyltransferase 2 (cytosolic)	Fw: TGTCACAGAACAGGGCAGAG<br>Rev: TGACAGTTCCTGTCCCATCA
Acly	ATP citrate lyase	Fw: TTCCTCCTTAATGCCAGCGG<br>Rev: GACTTGGGACTGAATCTTGGGG
Acss2	Acetyl-coenzyme A synthetase, cytoplasmic	Fw: CTGAGTGGATGAAAGGAGCAAC<br>Rev: CAGGAGTTCACGGTATGTGATC
Adk	Adenosine kinase	Fw: CTATGCTGCCGAGAACAACA<br>Rev: TGCCTCTTCGAGTTCACCTT
Ampd3	Adenosine monophosphate deaminase 3	Fw: AGGAGCTTGCAGAGCAGAAG<br>Rev: GATGGTGACCCGCTGATACT
Apoa1	Apolipoprotein A-I	Fw: CTTGGCACGTATGGCAGCA<br>Rev: CCAGAAGTCCCGAGTCAATGG
Apoa4	Apolipoprotein A-IV	Fw: AGGGACACAGGTACACCGTT<br>Rev: AGTTTGTCCTGGAAGAGGGTACTGA
Arbp	Ribosomal protein, large, P0 (*Rplp0*)	Fw: TTTGGGCATCACCACGAAAA<br>Rev: GGACACCCTCCAGAAAGCGA
Bcat2	Branched-chain amino acid aminotransferase 2 (mitochondrial)	Fw: AGCAGGAGCTGTTGGAGTGT<br>Rev: ATGACGTACAGGAGGGCTTG
Bckdha	2-oxoisovalerate dehydrogenase subunit alpha, mitochondrial (Branched-chain keto acid dehydrogenase E1 subunit alpha)	Fw: ACATTGGGCTGGATGAACTC<br>Rev: CCGAAATCACCACGACACCT
Cebpa	CCAAT/enhancer binding protein alpha	Fw: AGTCGGTGGACAAGAACAGC<br>Rev: GTCACTGGTCAACTCCAGCA
Cmpk1	Cytidine monophosphate (UMP-CMP) kinase 1	Fw: CCTCTCCACCTCATGAAGCC

		Rev: TGTGTGTAGCCGTATTTCTCG
Cpa3	Carboxypeptidase A3, mast cell	Fw: AATTGCTCCTGTCCACTTTGAC<br>Rev: TCACTAACTCGGAAATCCACAGT
Cidea	Cell death-inducing DNA fragmentation factor alpha	Fw: ATCACAACTGGCCTGGTTACG<br>Rev: TACTACCCGGTGTCCATTTCT
Dlat	Dihydrolipoyl transacetylase	Fw: TCCGCATCAGAAGGTTCCATT<br>Rev: CCAGGCTCTCAAACCCAACA
Hk1	Hexokinase 1	Fw: ACGACACCCCAGAGAACATC<br>Rev: TTCAGCAGCTTGACCACATC
Hprt	Hypoxanthine guanine phosphoribosyl transferase	Fw: AGTCCCAGCGTCGTGATTAG<br>Rev: TGATGGCCTCCCATCTCCTT
Idh1	Isocitrate dehydrogenase 1 (NADP+), soluble	Fw: AGGTTCTGTGGTGGAGATGC<br>Rev: CGCCCACGTTGTATTTCTTT
Impdh2	Inosine monophosphate dehydrogenase 2	Fw: CAGGCATGGCTTCTGTGGTA<br>Rev: GCCAACATATAGCAGCCCGAG
Mcpt4	Mast cell protease 4	Fw: TAGACCACATTCTCGCCCTTA<br>Rev: GGATTCTGTCTTGCTCACATCA
Nadk	NAD kinase	Fw: ATTATGCTGTCACCGGAAGC<br>Rev: GCTGTCCTCCTCATCCTCTG
Naprt	Nicotinate phosphoribosyltransferase	Fw: TGCCCTGGCTAGAGTCTGTT<br>Rev: TGCTTCTCAGGGTCCTCTGT
Nme1	NME/NM23 nucleoside diphosphate kinase 1	Fw: GCGGTAAAGCCTTGTCATCTG<br>Rev: CCCAGACCATAGCAACCACT
Nme3	NME/NM23 nucleoside diphosphate kinase 3	Fw: TCTGGTGCTGACCATCTTTG<br>Rev: CACTAGCTTCAGTGCCACCA
Nudt5	Nudix hydrolase 5 (ADP-sugar pyrophosphatase 5)	Fw: GGAAGAAACTGGCTACAAAGGT<br>Rev: TTGGCCTTACATTTCCTGCAT
Paics	Phosphoribosylaminoimidazole carboxylase	Fw: CAGGGAATGCAGCTAGGAAG<br>Rev: TGAGGAAAGACCCAGTTGCT
Pck2	Phosphoenolpyruvate carboxykinase 2, mitochondrial	Fw: CTCAACTCCCTCTGGCTTTG<br>Rev: ATGCAGGGTTTGGATGCTAC
Pde4d	Phosphodiesterase 4D, cAMP specific	Fw: AGAGCCTGTCTTGTAAGCATTTC<br>Rev: GGTGCGGCTCTCAGTAGTTT
Pde8b	Phosphodiesterase 8B	Fw: CAGCGTCAAGCAGGTGTCT<br>Rev: GCTTGTGAAACGACAGCCAG
Pfas	Phosphoribosylformylglycinamidine synthase	Fw: AGACCTTCCCGAGAACCTTG<br>Rev: TGTACCTCCAACACCAGACC
Pgam	Phosphoglycerate mutase	Fw: GGCATTGTGAAACATCTGGAA<br>Rev: AACCTCATGGGCTTGGTG
Pgm2	Phosphoglucomutase 2	Fw: TGGCTGCGACTGCATTTATCA<br>Rev: GACACGGTGTACGGCACAAA
Pparg	Peroxisome proliferator-activated receptor gamma	Fw: TCAGCTCTGTGGACCTCTCC<br>Rev: ACCCTTGCATCCTTCACAAG
Pnpo	pyridoxine 5'-phosphate oxidase	Fw: AACTACTTCCATTCCCGCCC<br>Rev: CAGGATGTAGCCACCCCAGT
Prps1l3	Phosphoribosyl pyrophosphate synthetase 1-like 3	Fw: AAACCAAACCCACCCCTTGT<br>Rev: AGACCAAATTGTTTAGCAGAAATGT
Serpina3n	Serine (or cysteine) peptidase inhibitor, clade A, member 3N	Fw: CCCTGAGGAGTGGAAGAAT<br>Rev: CCTGATGCCCAGCTTTGAAA
Serpinb6	serine (or cysteine) peptidase inhibitor, clade B, member 6a	Fw: TGATACATCGCTAGTCCTTGTGA<br>Rev: ACAGGTTTCTCCTCATTCTTGC
Ucp1	Uncoupling protein 1	Fw: CAAATCAGCTTTGCCTCACTC<br>Rev: TAAGCCGGCTGAGATCTTGT
Tbp	TATA box binding protein	Fw: ACGCTTCACCAATGACTCCTA<br>Rev: TGACTGCAGCAAATCGCTTGG
